# Zirconium-Based Metal–Organic Frameworks as Acriflavine Cargos in the Battle against Coronaviruses—A Theoretical and Experimental Approach

**DOI:** 10.1021/acsami.2c06420

**Published:** 2022-06-14

**Authors:** Przemysław J. Jodłowski, Klaudia Dymek, Grzegorz Kurowski, Jolanta Jaśkowska, Wojciech Bury, Marzena Pander, Sylwia Wnorowska, Katarzyna Targowska-Duda, Witold Piskorz, Artur Wnorowski, Anna Boguszewska-Czubara

**Affiliations:** † Faculty of Chemical Engineering and Technology, Cracow University of Technology, 24 Warszawska, 31-155 Kraków, Poland; ‡ Faculty of Chemistry, University of Wrocław, 14 F. Joliot-Curie, 50-383 Wrocław, Poland; § Department of Medical Chemistry, Medical University of Lublin, 4A Chodzki, 20-093 Lublin, Poland; ∥ Department of Biopharmacy, Medical University of Lublin, 4A Chodzki, 20-093 Lublin, Poland; ⊥ Faculty of Chemistry, Jagiellonian University, Gronostajowa 2, 30-387 Kraków, Poland

**Keywords:** metal−organic frameworks, acriflavine, SARS-CoV-2, drug delivery, density functional theory calculations

## Abstract

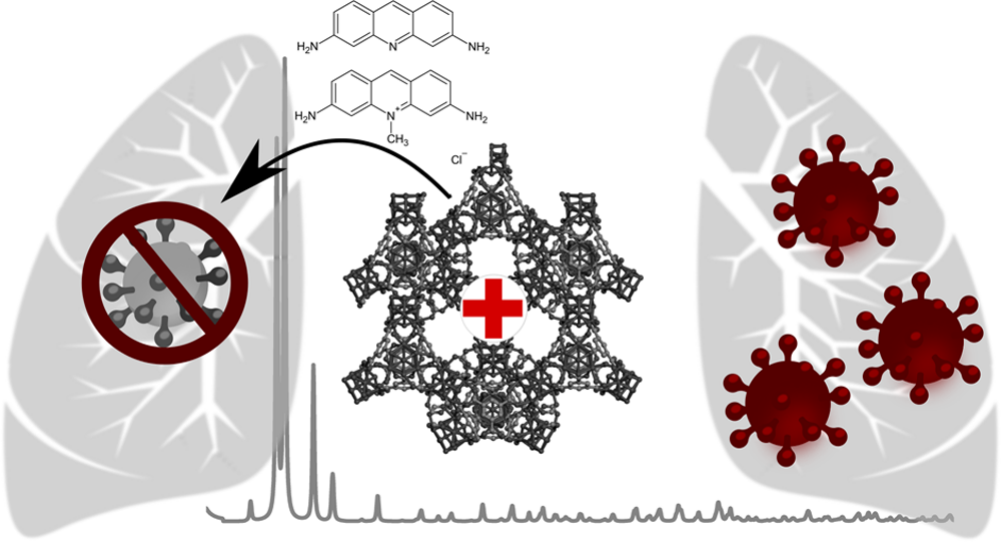

In this study, we present a complementary approach for obtaining an effective drug, based on acriflavine (ACF) and zirconium-based metal–organic frameworks (MOFs), against SARS-CoV-2. The experimental results showed that acriflavine inhibits the interaction between viral receptor-binding domain (RBD) of spike protein and angiotensin converting enzyme-2 (ACE2) host receptor driving viral cell entry. The prepared ACF@MOF composites exhibited low (MOF-808 and UiO-66) and high (UiO-67 and NU-1000) ACF loadings. The drug release profiles from prepared composites showed different release kinetics depending on the local pore environment. The long-term ACF release with the effective antiviral ACF concentration was observed for all studied ACF@MOF composites. The density functional theory (DFT) calculations allowed us to determine that π–π stacking together with electrostatic interaction plays an important role in acriflavine adsorption and release from ACF@MOF composites. The molecular docking results have shown that acriflavine interacts with several possible binding sites within the RBD and binding site at the RBD/ACE2 interface. The cytotoxicity and ecotoxicity results have confirmed that the prepared ACF@MOF composites may be considered potentially safe for living organisms. The complementary experimental and theoretical results presented in this study have confirmed that the ACF@MOF composites may be considered a potential candidate for the COVID-19 treatment, which makes them good candidates for clinical trials.

## 1 Introduction

The COVID-19 pandemic caused by the SARS-CoV-2 virus, since its first reports in China in 2019, generated over 400 million cases of illness and claimed nearly 6 million lives according to the Johns Hopkins Coronavirus Resource Center (CRC).^1^ The invention and implementation of a vaccination program worldwide changed the course of the pandemic by reducing the total number of cases and consequently suppressed the number of hospitalizations and deaths.^2^ To date, several treatment strategies have been developed for patients with COVID-19 or immunocompromised patients who cannot be vaccinated. Depending on the patient’s condition, treatment includes the application of dexamethasone, glucocorticoids, tocilizumab, or remdesivir in doses, and the period of admission selected individually.^2,3^ Recently, for moderately or severely immunocompromised patients, the Infectious Diseases Society of America (IDSA) recommends prophylactic treatment with tixagevimab/cilgavimab. Apart from recommendations, the development of new active drugs to inhibit the growth of the SARS-CoV-2 virus is highly demanded. Recently, several routes of development of therapeutic agents considering the SARS-CoV-2 lifecycle and the host–virus interactions were reported.^4−6^ The enormous interest in the development of a new active drug for covid treatments has resulted in the development of new methods of drug design based on neural networks, which, compared to traditional methods of drug design, allow for a significant reduction in research time.^5^ In the work by Amilpur and Bhukya,^5^ the model based on leverages on long short-term memory was developed that allows for the generation of molecules that potentially bind with SARS-CoV-2 protease.

In a recent work by Napolitano et al.,^7^ acriflavine (ACF)—a mixture of 3,6-diamino-10-methylacridinium chloride (trypaflavine) and 3,6-diaminoacridine (proflavine)—was proposed as a potential drug that effectively inhibits β-coronaviruses including SARS-CoV-2. In their work, a high-throughput screening indicated that acriflavine shows papain-like protease inhibition and additionally blocks papain-like protease catalytic pockets. The nanomolar inhibitory activity of the drug toward the viral replication of SARS-CoV-2 was confirmed in cellular models and *in vivo*. Additionally, the low cost of ACF may increase its availability to patients from low- and lower-middle-income countries.

**Figure 1:**
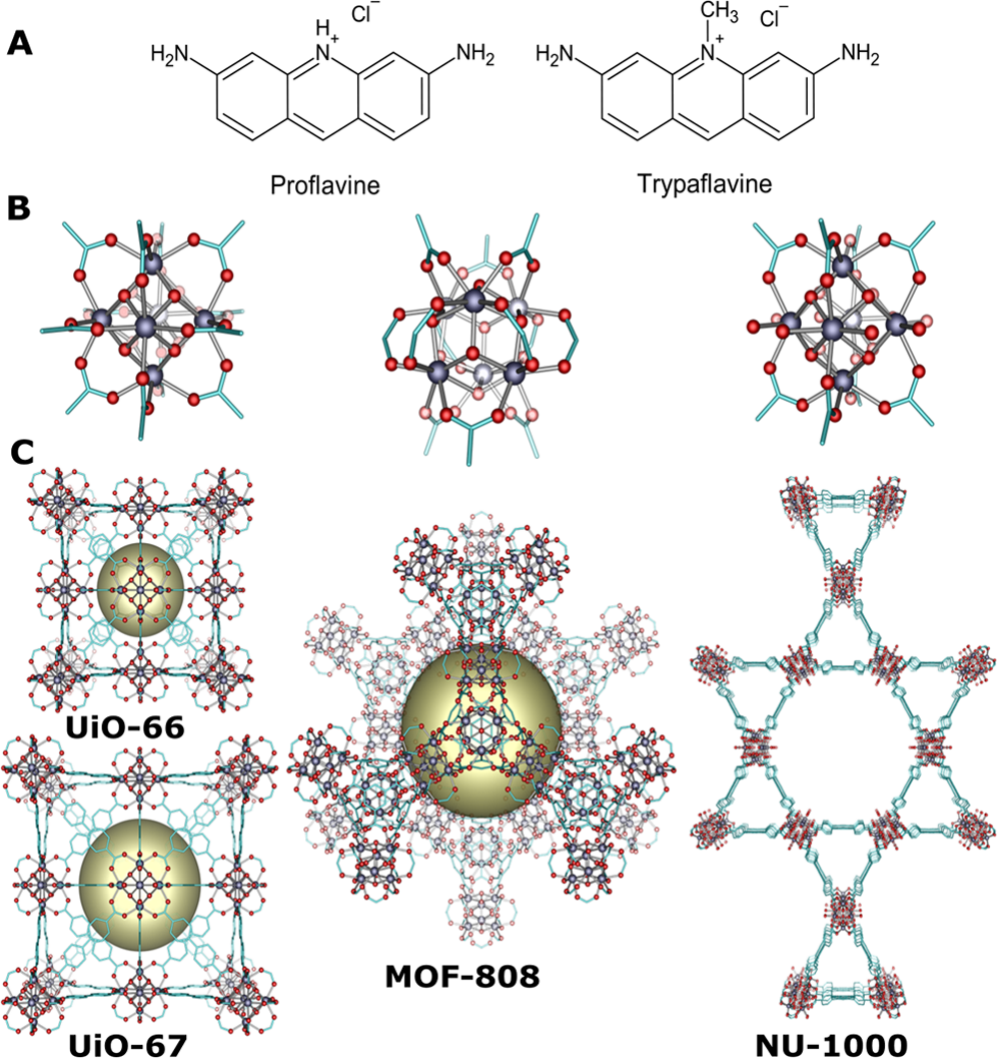
(A) ACF structure (3,6-diamino-10-methylacridinium hydrochloride and 3,6-diaminoacridine hydrochloride); (B) types of Zr_6_ nodes in Zr-based metal–organic frameworks; and (C) view of the frameworks for MOFs presented in this work; yellow spheres demonstrate the largest cages; hydrogen atoms have been omitted for clarity.

Apart from the development of novel active drugs, it is necessary to discover efficient and safe methods for their administration that limit side effects and reduce the engagement of medical personnel. In this context, metal–organic frameworks (MOFs), a class of porous materials, have been regarded as attractive carriers for drug delivery. Since their first use as drug cargo in 2006,^8^ they have been experiencing rapid growth in medical applications. Numerous studies reported their use in the delivery of ibuprofen,^8^ 5-fluorouracil,^9,10^ oridonin,^11^ anethole,^12^ and chloroquine.^13^ Among many advantages of using MOFs as drug carriers, the most important are the controlled release of the drug, which can be optimized at the design stage of the MOFs, and indirectly reducing the side effects of drugs, and the protective nature of MOFs in the case of administering biomolecular drugs.^14^ In our recent work,^13^ we reported the use of defective UiO-66 as chloroquine cargos. Based on *in vitro* and *in vivo* experiments, we showed that proper framework optimization during the preparation step results in prolonged chloroquine delivery and, what is more important, elimination of chloroquine side effects. Among the wide group of metal–organic frameworks, Zr-based MOFs are of great interest due to their high surface area, stability, a wide range of applications, versatility, and postmodification possibilities. The use of MOFs as drug cargos requires high biocompatibility, biodegradability, and low cytotoxicity for living cells.^8,14−16^ Of a large number of MOFs that have been obtained over the last two decades, Zr-based MOFs have the properties necessary for drug delivery systems (DDS). In this study, we chose four model materials: MOF-808,^17^ UiO-66,^18^ UiO-67,^18^ and NU-1000^19^ that meet the requirements for DDS. The structures of selected Zr-MOFs and their topologies are shown in Figure 1.

Based on our previous experience, and facing new challenges in the development of novel effective drugs for COVID-19 treatment, we wish to answer the following questions: (a) whether the group of zirconium-based MOFs may be potential candidates for ACF delivery, (b) is there any antiviral potential of prepared ACF@MOF composites, and (c) are ACF@MOF composites safe for cells and living organisms?

Considering the above-mentioned problems, in this work, we aim at a comprehensive understanding of structure–drug delivery–drug efficiency interactions in the preparation for potential ACF delivery systems based on MOFs. Our work demonstrates a multidisciplinary approach considering material preparation, modification, drug release, molecular modeling, as well as *in vitro* and *in vivo* experiments.

## 2 Experimental Section

### 2.1 Material Syntheses and Characterization

Zirconium-based metal–organic frameworks (Zr-MOFs), namely, MOF-808,^20^ UiO-66,^18^ UiO-67,^18^ and NU-1000,^19^ were synthesized following literature procedures. The detailed synthesis protocols for MOFs and ACF@MOF composites are provided in the Supporting Information. The synthesized materials were characterized by powder X-ray diffraction (PXRD, Figure S1), cryogenic adsorption of N_2_ (Figures S2–S4), scanning electron microscopy (SEM, Figure S5), ^1^H nuclear magnetic resonance (NMR) spectroscopy (Figures S6–S10) and attenuated total reflectance Fourier transform infrared (ATR-FTIR) spectroscopy. Detailed information about the characterization methods used is provided in the Supporting Information.

### 2.2 Acriflavine Release Profiles

The acriflavine release profiles from prepared ACF@MOF composites were performed according to the procedures described in our previous work^13^ with some modifications. In brief, 10 mg of ACF@MOF composites were placed in a 10 mL liquid medium (H_2_O, 10 mM PBS pH = 5.5). The ACF release kinetics was measured under thermostatic conditions set to 36.6 °C. The amount of the acriflavine release from ACF@MOF composites was monitored by ultraviolet–visible (UV–vis) spectroscopy at 450 nm using the AvaSpec ULS3648 spectrometer equipped with a transmission cuvette holder and a Ocean Optics DH-2000-BAL UV–vis–NIR light source.

### 2.3 Periodic Density Functional Theory (DFT) Calculations

The electronic structure DFT calculations, to determine the adsorption sites of guest molecules (ACF) in prepared composites, were performed with the use of the VASP^21,22^ code. A detailed description of the methodology and the models used are provided in the Supporting Information file. The geometrical relationship between the aromatic platform of the ACF molecules and the aromatic rings of the MOF hosts was described in the spirit of their π–π orbital. The geometric descriptors have been determined by the analysis of the average (least square fit) planes of the aromatic rings.

### 2.4 Protein–Protein Interaction Assay

The strength of the interaction between the viral RBD and human ACE2 was assessed using Lumit SARS-CoV-2 Spike RBD:hACE2 immunoassay (Promega) according to the manufacturer’s protocol optimized to 384-well plates. The assay is based on the immunodetection and complementation of a reporter enzyme (*i.e*., luciferase). In brief, our compound of interest or vehicle was incubated for 30 min with rabbit Fc-domain tagged RBD. Next, mouse Fc-domain tagged ACE2 was added followed by the addition of a secondary antibody cocktail composed of anti-rabbit and anti-mouse antibodies tethered with two complementary parts of the luciferase enzyme. The components of the mixture were allowed to interact for 60 min at room temperature. Then, luciferase substrate was added and the luminescent signal was recorded 30 min later using Synergy H1 multimode plate reader (BioTek).

### 2.5 Molecular Docking Simulations

The available cryo-EM structure of human triple ACE2-bound SARS-CoV-2 trimer spike at 3.64 Å atomic resolution (PDB ID: 7KMS)^23^ was used to perform molecular docking. For the molecular docking procedure, 3,6-diaminoacridine and 3,6-diamino-10-methylacridinium structures were downloaded from the PubChem database (PubChem CID 443101) and subsequently optimized using the LigPrep protocol from the Schrödinger suite of software v. 2020-3 (Schrödinger release 2020-3, Maestro, Schrödinger, LLC, New York, 2020). To sample ligand protonation states at physiological pH, the Epik module from the Schrödinger suite was also applied, as previously described.^24^ AutoDock Vina^25^ was subsequently used for docking simulations of flexible molecules into RBD of SARS-CoV-2 spike protein. The chosen dimension for the grid maps was 40 Å × 40 Å × 40 Å, with a grid-point spacing of 1 Å, to cover the RBD domain with the RBD-ACE2 interface. The parameters used with AutoDock Vina were the number of modes (20) and exhaustiveness (570). The energetically lower poses were selected from each cluster of superposed poses for either 3,6-diaminoacridine or 3,6-diamino-10-methylacridinium at each binding site and are presented in Table S7.

### 2.6 Cytotoxicity

A human fibroblast (ATCC: CRL-2522) and a rat H9C2(2-1) cardiomyoblasts (ATCC:CRL-1446; RRID:CVCL_0286) were used to evaluate the cytotoxicity of ACF as well as MOFs and ACF@MOF composites. The BJ cells were cultured in EMEM supplemented with 10% of fetal bovine serum (FBS), penicillin (100 U/mL), and streptomycin (100 μg/mL). The H9C2 myoblast was maintained in Dulbecco’s modified Eagle’s medium (DMEM) supplemented with FBS (10%), penicillin (100 U/mL), and streptomycin (100 μg/mL). Both cell lines were incubated at 37 °C in a humidified atmosphere of 5% CO_2_. Every 3 days, the cells were fed and subcultured to prevent cell differentiation.

For the experiments, cells between passages 7 and 20 were used. First, the cells were seeded (5 × 10^5^ cells/mL) on 96-well plates and incubated with ACF for a period of 24 h in a concentration ranging from 0–50 μg/mL (0 μM to 200 mM). Then, cell viability was evaluated using the (3-(4,5-dimethylthiazol-2-yl)-2,5-diphenyltetrazolium bromide) (MTT). IC50 for ACF was determined.

Having established the toxic range of ACF for the BJ and H9C2 cell lines, a similar MTT test was performed for MOFs and ACF@MOF composites. The images were taken with the use of a Leica DMi1 microscope.

### 2.7 Ecotoxicity

To determine the ecotoxicity of acriflavine, the fish embryo toxicity (FET) test was performed on zebrafish (*Danio rerio*) according to OECD Test Guideline 236 in a procedure described before.^13^ At the end of the exposure period (96 hpf—hours postfertilization), acute toxicity was determined based on a positive outcome in any of the four visual indicators of lethality, including the coagulation of fertilized eggs, lack of somite formation, lack of detachment of the tailbud from the yolk sac and lack of heartbeat. The value of LC_50_ was calculated. Heartbeats were recorded. Moreover, images of the fish from each group were taken at the final time point to monitor the occurrence of developmental malformations. For the observations, a Discovery V8 Stereo optical microscope and Zeiss hardware were used.

### 2.8 Statistical Analysis

The data statistics for ACF release profiles were performed using Prism 9 (GraphPad Software) by fitting the experimental data and calculating SEM (standard error of the mean) values.

Differences in protein:protein interaction strength in samples subjected to either vehicle or increasing concentrations of acriflavine were assessed using an estimation statistics framework and presented on a Cumming estimation plot.^26^ The effect sizes and CIs were reported as *effect size* [*CI width lower bound*; *upper bound*]. 5000 bootstrap samples were taken; the confidence interval was bias-corrected and accelerated. The *p*-values reported are the likelihoods of observing the effect sizes if the null hypothesis of zero difference is true. For each permutation *p*-value, 5000 reshuffles of the control and test labels were performed.

A dose–response curve was generated using Prism 9 (GraphPad Software) by fitting a biphasic sigmoid curve model to experimental datapoints. The concentrations of the compound of interest causing 50% inhibition (IC50) of the protein–protein interaction were calculated.

## 3 Results and Discussion

### 3.1 Synthesis and Characterization of Drug Cargos

For the proper design of DDS, the structural features of selected MOFs have to be carefully analyzed. All MOFs, selected for this study, are composed of Zr_6_-oxo-clusters bridged by polytopic linkers. The resulting MOF structures represent different topologies, pore sizes, and pore geometries, *e.g.*, UiO-66 and its isoreticular analog UiO-67, contain octahedral cages of 10.2 Å (14.4 Å) internal diameter connected by 5.9 Å (8.3 Å) triangular windows,^27^ MOF-808 contains large adamantane-like cage with an average diameter of 18 Å connected by small hexagonal windows with a diameter of 10 Å (Figure 1).^17^ Finally, NU-1000 build of tetratopic TBAPy^4–^ linkers (where TBAPy = 1,3,6,8-tetrakis(p-benzoate)pyrene), contains large (30 Å) one-dimensional channels.^19^ It should be noted that UiO-66 used in this study was prepared using concentrated hydrochloric acid as a modulator.^28^ Such an approach yields a highly defective material, in which an average channel and cage diameter differ from the “defect-free” UiO-66 sample,^29^ with a considerably larger number of pores within the 20–100 Å range. The modulated synthesis was under intense investigation for various Zr-MOF structures including UiO-66,^30−32^ UiO-67,^33^ and MOF-808.^34^ In work by Shearer et al.,^31^ structural defects in UiO-66 were deeply investigated, and more recently, the nature of the defects in UiO-66 structure was examined by HRTEM microscopy by Liu et al.^35^

The successful preparation of selected Zr-MOF materials and ACF@MOF composites was confirmed by powder X-ray diffraction (PXRD) (Figure S1). The comparison of simulated and experimental PXRD patterns confirmed the high crystallinity of the prepared materials for pristine MOFs. To confirm the porosity of pristine MOFs and ACF@MOF composites, N_2_ adsorption experiments were carried out at 77 K. The adsorption isotherms are shown in Figures S2 and S3, and the porosity parameters are summarized in Table 1. For MOF-808, UiO-66, and UiO-67, the type I adsorption isotherms were observed, and for NU-1000, characteristic type IV isotherm was obtained. These results are in good agreement with previous reports for zirconium-based MOF materials.^18−20,36^ When comparing the results of the low-temperature N_2_ sorption analyses, the decrease in porosity in prepared ACF@MOF composites may be observed for all samples. The decrease in porosity is correlated with the amount of introduced ACF into the MOF matrix and is most prominent for ACF@UiO-67 and ACF@NU-1000 samples, respectively.

**Table 1:** Sample Details

Sample	*S*_BET_, m^2^/g	Total pore volume, cm^3^/g	Acriflavine loading*, wt %	Acriflavine loading**, wt %	Simulated ACF loading, wt %
MOF-808, (ACF@MOF-808)	1454 (584)	0.821 (0.419)	4.13 ± 0.3	1.2	39.3
UiO-66, (ACF@UiO-66)	1421 (949)	0.685 (0.538)	5.55 ± 0.5	<0.5	0
UiO-67, (ACF@UiO-67)	2352 (278)	0.992 (0.153)	43.53 ± 0.002	36	38.0
NU-1000, (ACF@NU-1000)	2081 (259)	1.42 (0.155)	47.62 ± 0.01	37	53.6

* determined by UV–Vis spectroscopy.

** determined by ^1^H NMR spectroscopy; values in parentheses correspond to ACF@MOFs composites.

### 3.2 Acriflavine Loading—A Theoretical and Experimental Approach

To assess the theoretical acriflavine capacity of selected MOFs, Monte-Carlo (MC) simulations were performed. For each MOF, the number of ACF molecules at the equilibrium point per unit cell was calculated. The equilibrium loading was estimated by means of the MC simulations at 298 K, with the use of the Metropolis algorithm and Universal Force Field.^37^ The loadings were modeled for the fugacity equal to 100 kPa. The numerical results of MC simulations are shown in Table 1. The theoretical values, represented as number of ACF molecules per Zr_6_ node, are in good agreement with the calculated pore volumes, *i.e*., NU-1000 > UiO-67 > MOF-808 > UiO-66. For the determination of void volume, the Connolly Accessible Solvent Surface was simulated (initial solvent radius = 1.4 Å, maximal solvent radius = 2.0 Å), and the resultant void fraction correlated very well with the average Monte-Carlo number of ACF molecules per unit cell. The correlation coefficient, *R*^2^, was found to be equal to 0.81, with NU-1000 possessing proportionally higher sorption capacity than would have been expected with the same linear model developed for the remaining MOFs (in the latter case, *R*^2^ reaches the value of 0.93). Such behavior seems to stem from the advantageous shape of the pore system in NU-1000.

Moreover, the pore and aperture size in the MOF frameworks, together with the size of the ACF molecules, were also estimated based on the maximum fitting sphere approximations and the results (see Table S6) are in very good agreement with the experimentally derived pore size distribution (Figure S2).

To prepare ACF@MOF composites, activated samples of pristine MOFs were immersed in aqueous solutions of commercially available acriflavine source, which is composed of two major components: proflavine and acriflavine (as chloride salts). The encapsulation process was carried out under ambient conditions for 24 h. The subsequent ACF@MOF composite washing and separation (by centrifugation) were performed to minimize the effect of external ACF anchoring, which could blur the recognition of the adsorption sites in MOFs. The two-step procedure of composite preparation allows acriflavine to diffuse efficiently and homogenously into the MOF structure.

The ACF loadings in the prepared ACF@MOF composites were determined by UV–vis and ^1^H NMR spectral studies. The results are shown in Tables 1 and S1 and Figures S6–S10. The general observation is that ACF loading increases with the increasing pore volume of Zr-MOFs (cf. Table 1). The highest ACF loadings were achieved for NU-1000 and UiO-67, reaching 47.6 and 43.5 wt %, respectively. The lowest ACF loadings of 5.6 and 4.1 wt %, were obtained for UiO-66 and MOF-808, respectively. Additionally, to further confirm the ACF content in the ACF@MOF composites, we performed ^1^H NMR spectroscopy experiments for dissolved ACF@MOF samples (see Figures S6–S10 in the Supporting Information). Based on the comparison of intensities of corresponding signals from the linkers and ACF molecules, the content of ACF in ACF@NU-1000 and ACF@UiO-67 was estimated to be 37 and 36 wt %, respectively. Similarly, to UV–vis experiments, for ACF@MOF-808 and ACF@UiO-66, we observed very low intensities of ACF peaks (approx. 1 wt %). The obtained NMR results for ACF@MOFs are consistent with the results from UV–vis spectroscopy.

The MC simulated loadings agree very well (*R*^2^ = 0.994) with the experimental ones with exception of the MOF-808 structure, where the underestimation can be attributed to the presence of the formate anions, bonded to the nodal clusters, thus obstructing the openings of the cages. The rigid host approximation can rationalize the systematic underestimation of the modeled loading (*ca*. 0.68 of the experimental value, calculated from the linear regression) for the MOF structures with tight channels. Another source of the systematic discrepancies between the simulated and experimental loading can be traced to the generally understood imperfections of the crystal (with respect to the perfectly 3D translationally symmetrical computational model), *i.e*., the influence of the external surface of the crystallites and their defecting.

To comprehensively understand the mechanisms and interactions between the guest (ACF) and hosts (MOFs), we performed DFT calculations. The model geometries, sorption energies, and sorption geometries are given in Tables S2–S4, respectively. The initial positions of the ACF molecules in the MOF frameworks were found using the MC simulations within the rigid host approximation. The geometries were subsequently optimized at the DFT+D GGA level of theory (see Figures S12–S20, see Supporting CIF files). The differences between the energies of the same molecule in different unit cells were as high as *ca*. 0.4 eV (see Table S3). The most pronounced π–π stacking between host and guest is observed for NU-1000 (structure 1, both 3,6-diamino-10-methylacridine and 3,6-diaminoacridine) and UiO-66. In the case of structure 2 in NU-1000 (both 3,6-diamino-10-methylacridine and 3,6-diaminoacridine) the relative orientation of the aromatic platforms of the ACF molecules and the ligands was perpendicular and hence, for the reason of symmetry, π–π stacking was not possible. The lack thereof is also reflected in the sorption energy, where for structure 2, it was weaker by up to 0.35 eV (3,6-diamino-10-methylacridine@NU-1000).

### 3.3 Structural Characterization of ACF@MOF Composites

**Figure 2:**
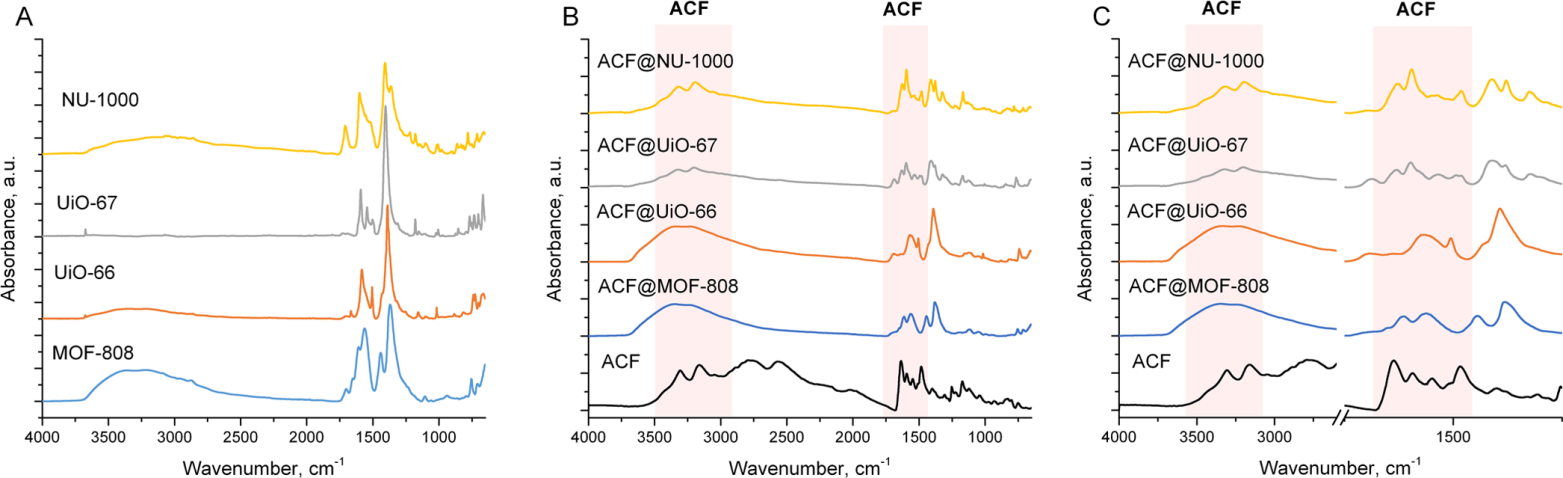
Spectroscopic characterization of the prepared materials: (A) ATR-FTIR spectra of pristine metal–organic frameworks; (B, C) ATR-FTIR spectra of ACF@MOF composites.

The ATR-FTIR spectra of pristine MOFs and ACF@MOF composites are shown in Figure 2. The pristine Zr-MOFs showed typical patterns which are in good agreement with the results described in the literature. The good agreement of the ATR-FTIR spectra supports the data obtained by PXRD. The spectra of pristine MOF-808, UiO-66, and NU-1000 materials exhibit a broad band in 3600–2600 cm^–1^, which correspond to the OH vibrations of adsorbed water molecules. The UiO-67 spectrum reveals an additional sharp band at 3675 cm^–1^ which is associated with isolated OH vibrations and weak bands in 3100–2850 cm^–1^ originating from −CH aromatic and aliphatic vibrations of benzene rings.^18^ The bands in the 1750–1000 region originate from Zr-MOF fingerprints which are associated with in-phase and out-of-phase carboxylate vibrations and carbocyclic and heterocyclic C=C stretching vibrations.^18^ The ATR-FTIR spectra of ACF@MOF composites confirm the successful incorporation of guest molecules into the MOF materials. The ACF characteristic bands (ACF markers, Figure 2B,C) at 3295 and 3157 cm^–1^ originate from N–H vibrations, whereas the bands at 1636, 1594, and 1483 cm^–1^ originates from C=N aromatic ring, C=C phenyl, C–H scissoring vibrations.^38,39^ The zoomed view on ATR-FTIR spectra of prepared composites (Figure 2C) shows that the ACF characteristic bands become evident in ACF@UiO-66, ACF@UiO-67, and ACF@NU-1000 composites.

**Figure 3:**
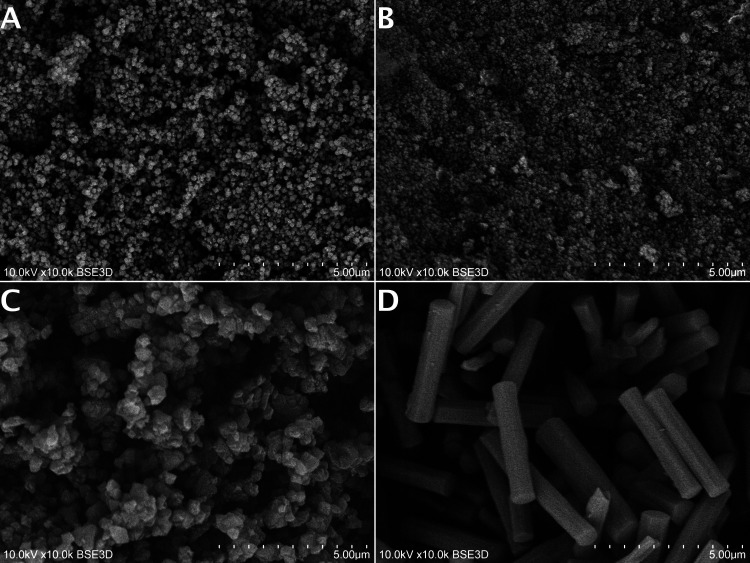
SEM images of synthesized ACF@MOF composites: (A) ACF@MOF-808, (B) ACF@UiO-66, (C) ACF@UiO-67, (D) ACF@NU-1000.

The morphology of prepared pristine and ACF@MOF composites was determined by SEM microscopy (Figures 3 and S5). The morphology of pristine materials was in good agreement with the literature data.^20,40^ The MOF-808 shows well-defined cubic nanocrystals with *ca*. 250 nm particle size. Similar observations were made for the UiO-66 sample (Figure 3B), where aggregated oval *ca*. 200 nm crystals were formed. The SEM analysis of UiO-67 (Figure 3C) reveals the presence of MOF nanocrystals of 300 nm in diameter. The largest crystals from the selected group of Zr-MOF materials were determined for NU-1000. The NU-1000 shows uniform cylindrical microcrystals with an average length of 3 μm.

### 3.4 Analysis of Acriflavine Release from ACF@MOF Composites

**Figure 4:**
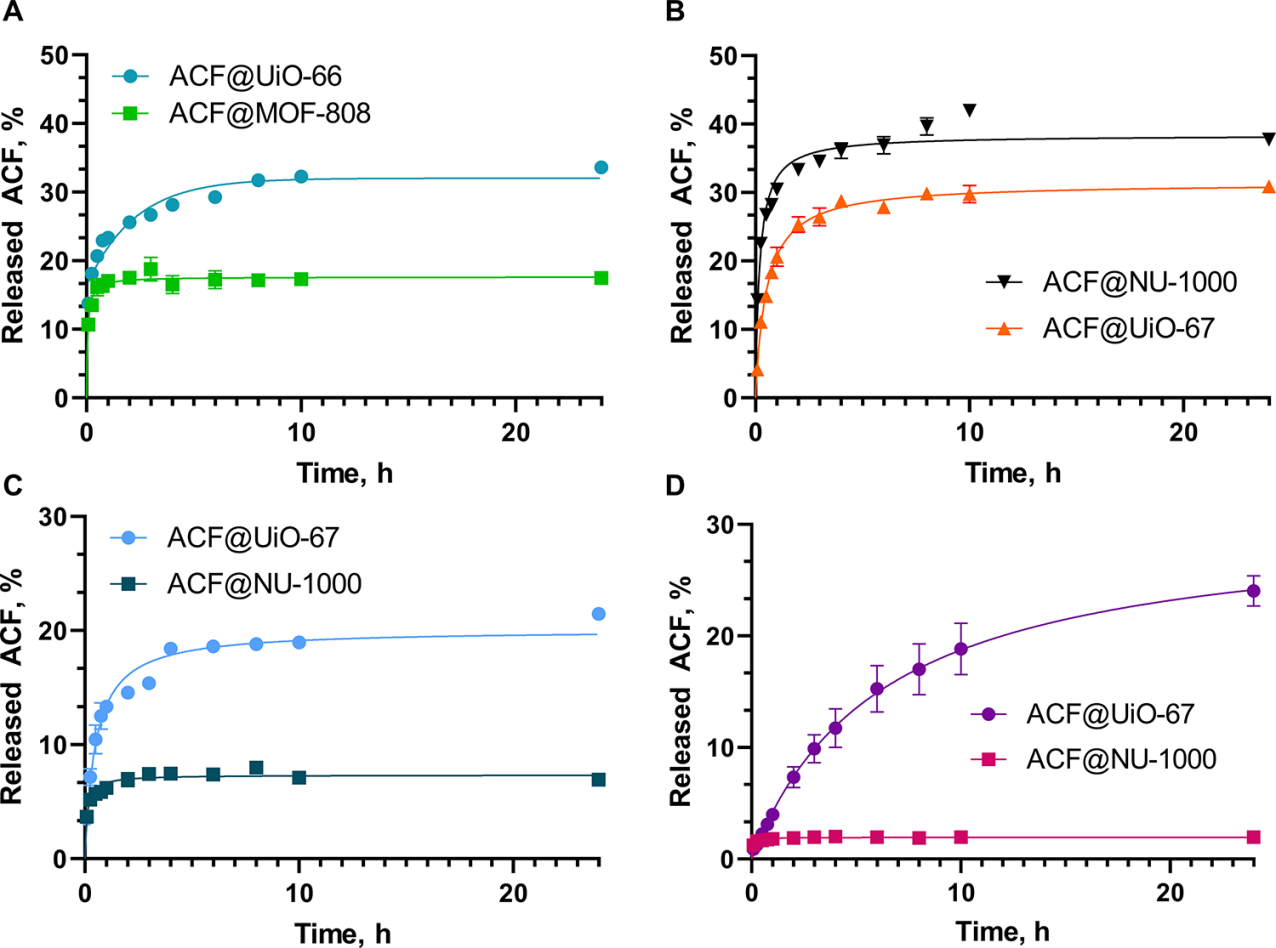
Acriflavine (ACF) release profiles for prepared composites: (A), (B) deionized water, (C) PBS pH = 5.5, (D) SBF.

Initially, acriflavine release profiles were examined in distilled water at 36.6 °C. The ACF release profiles from prepared ACF@MOF composites are shown in Figures 4 and S11. For low-loading ACF@MOF composites (ACF@MOF-808 and ACF@UiO-66), the lowest amount of ACF was released from ACF@MOF-808 reaching 17.5% ACF release, whereas for ACF@UiO-66, this value was around 35%. The high-ACF-loading samples characterize considerable ACF release of around 39 and 30% for ACF@NU-1000 and ACF@UiO-67 samples, respectively. All prepared ACF@MOF composites showed comparable release profiles. The vast majority of ACF molecules were released during the first 2 h and a clear plateau was achieved during 24 h.

The ACF release was further studied for the two most promising composites, UiO-67 and NU-1000 in the acidic PBS solution (Figure 4C) and in the SBF solution (Figure 4D). The general decrease in ACF release from prepared composites was observed in both solutions. The ACF release from ACF@UiO-67 decreased to 30% at 24 h, while a significant decrease to 3% release was observed for ACF@NU-1000. The most spectacular results were observed in the SBF solution for NU-1000, where only 2% of ACF encapsulated in the prepared composite was observed. The interesting results were determined for ACF@UiO-67 in the SBF solution. The total amount of ACF released was kept at the same level in comparison with acidic PBS solution, however, the release profile is smoother and stretched over time. We did not observe a rapid ACF release in the first 2 h, but we observed a steady release until it reaches its maximum value in 24 h. The decrease in the total amount of guest molecule release from zirconium-based MOFs was previously described elsewhere.^41,42^ In work by Jarai et al.^41^ the release of rhodamine B (RhB) from defective UiO-66 samples varied with the defect concertation (1, 8, 12, 15% defective). A considerable decrease in RhB form prepared UiO-66 composites was observed in artificial lysosomal fluid (ALF) and SBF fluid. The differences in RhB released from all prepared defective UiO-66 samples reached 90% of RhB release in ALF and SBF fluids. They concluded that in a more acidic medium the ligands become protonated and, as a result, are displaced by the component of SBF. Following this way of thought, in our experiments, ACF should exhibit enhanced release in acidic PBS solution in comparison with deionized water. On the contrary, in work by Jiang et al.^42^ the comparative release of diclofenac sodium (DS) was determined for two Zr-based MOFs—ZJU-808 (isoreticular to NU-801) and ZJU-801. The release of DS was determined at various pH = 7.4, 5.4, and 2.0. They noted decreased DS release from DS@ZJU-808 composites with decreasing pH value. Since the DS release at pH = 7.4 was close to 100%, as the pH decreased to 2.0, the DS was not released from prepared ZJU-808 composites. Unlike for ZJU-808, the reversed tendency was observed for DS@ZJU-801 samples. They concluded that the enhanced DS release under acidic conditions was due to electrostatic interactions between MOFs and guest molecules due to the protonation of N-linker sites. In our study, none of the selected Zr-MOF structures contain nitrogen sites; however, both 3,6-diaminoacridine and 3,6-diamino-10-methylacridinium contain nitrogen sites in which 3,6-diamino-10-methylacridinium is protonated.

For modeling the drug release, two equations were used: the first-order kinetics and the Gallagher–Corrigan (GC) model.^43^ The first one follows the formula
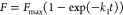
1while the GC model can be expressed as follows
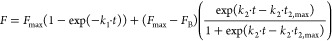
2where *F* is the fraction of drug released at a time *t*, *F*_max_ is the maximum fraction of drug released during the total time, and *k*_1_ and *k*_2_ are the first-order rate constants for the first and second stages, respectively. *F*_B_ denotes the drug fraction released in the first release stage.

The fitting of the experimental data to the model equations has been performed with the use of the Mathematica^44^ code, *via* the NonlinearModelFit function (see Table S5). It can be seen that the highest amount of the drug was released in water from NU-1000 and UiO-67, while the released amount for MOF-808 and UiO-66 was an order of magnitude lower. The relationship present for the former two frameworks is inverted in the environment of PBS and SBF. The release rate (water) was the highest for MOF-808 and NU-1000. In the environment of PBS and SBF, however, the release rate is much lower in the case of NU-1000, it drops *ca*. 2× and 10×, respectively, compared to the water phase. The ordering is inverse for UiO-66, when changing from water to SBF, the release rate increases from 7.4 to 68.3 μmol/dm^3^.

**Figure 5:**
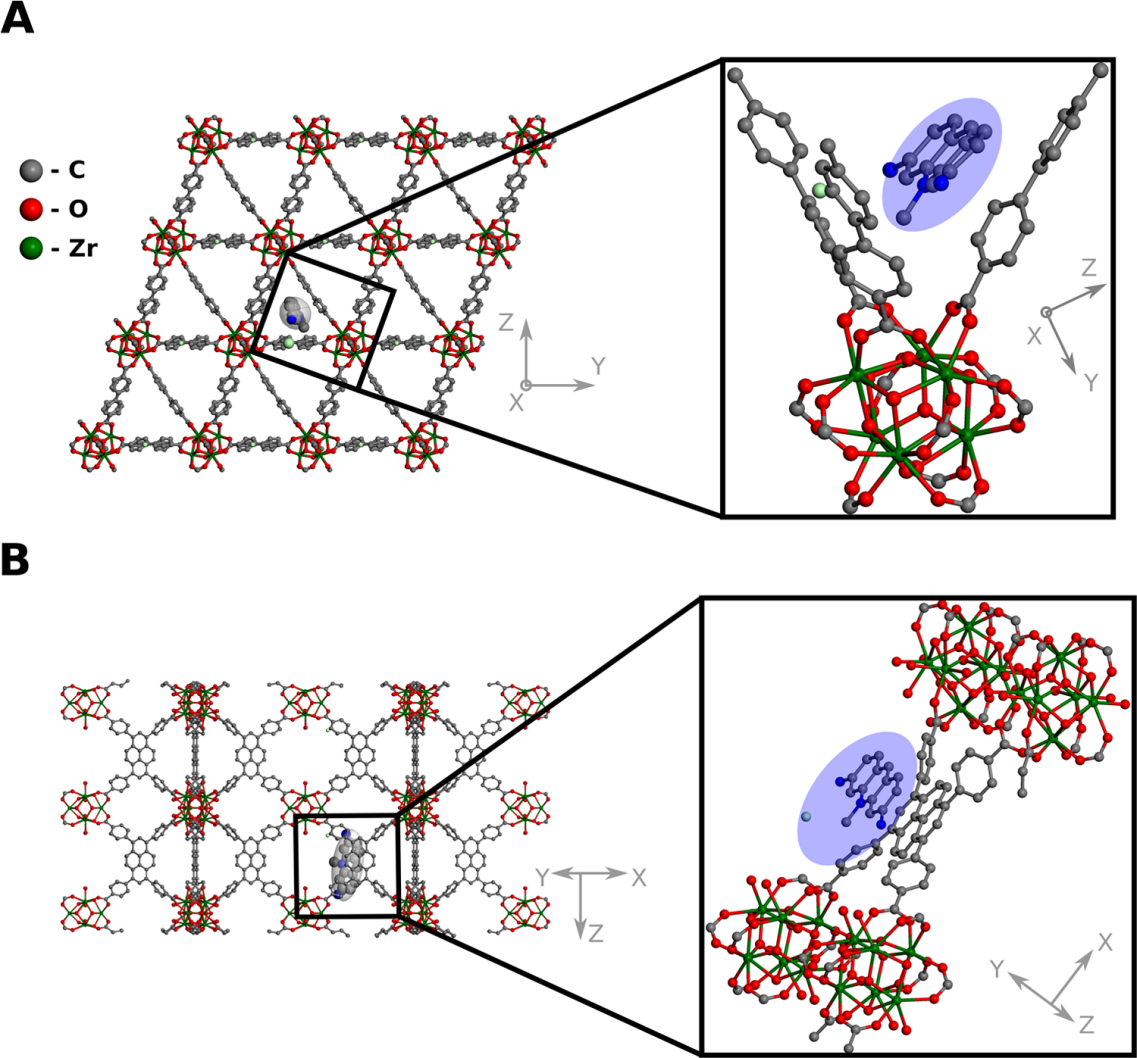
DFT-optimized structure of (A) UiO-67 and (B) NU-1000, with Zr_6_ nodes (zoomed area) with adsorbed 3,6-diamino-10-methylacridine emphasized with ellipsoid; hydrogens omitted for clarity; additional structures are provided in the Supporting Information.

The experimental release rates agree very well with computational (DFT) adsorption energies (Table S4, Figure 5), namely, for MOF-808 and NU-1000, the release is the fastest among the studied structures (rates: *ca*. 4.8 and 4.7 min^–1^, respectively) and the adsorption is the weakest (*ca*. −0.7 and −0.6 eV, respectively). For the other MOFs, UiO-66, and UiO-67, the rates are *ca*. 0.4 and 0.9 min^–1^, respectively, and the adsorption is much stronger; the adsorption energies are *ca*. −1.9 and −1.0 eV, respectively.

Noteworthy, the mechanism of the drug release from UiO-66 is well modeled by the first-order kinetics, while for UiO-67 and NU-1000, the two-stage Gallagher–Corrigan model is better applicable.

### 3.5 Acriflavine Inhibits Interactions between Viral RBD Domain of Viral S Protein with Its Human Receptor

**Figure 6:**
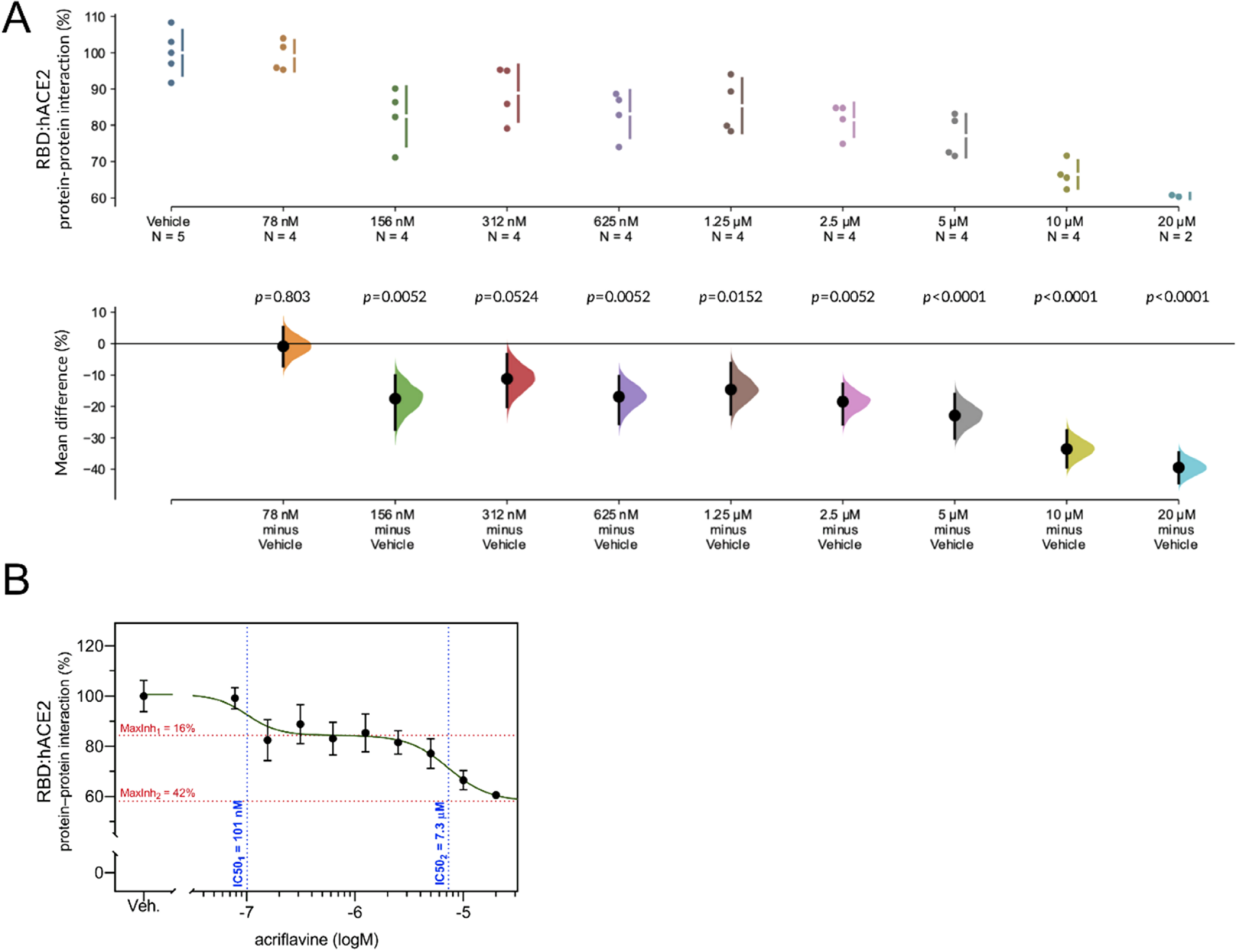
Acriflavine inhibits the interaction between viral RBD domain and human ACE2. (A) Interaction strength between the RBD domain of SARS-CoV-2 Spike protein and ACE2 was measured in the presence of increasing concentrations of acriflavine (starting from 78 nM up to 20 μM). The mean differences in the protein:protein interaction for nine acriflavine concentrations against the shared vehicle control (DMSO) are shown in the above Cumming estimation plot. The interaction values expressed as the percentage of signal detected in vehicle control samples are plotted on the upper axes. On the lower axes, mean differences are plotted as bootstrap sampling distributions. Each mean difference is depicted as a dot. Each 95% confidence interval is indicated by the ends of the vertical error bars. The *p*-values are reported above respective sampling distributions. (B) RBD:ACE2 interaction data were fitted to a biphasic dose–response curve. The binding of RBD to ACE2 was inhibited by *ca*. 16% with IC50_1_ = 101 nM, and by *ca.* 42% with IC50_2_ = 7.3 μM.

Acriflavine was recently reported to inhibit papain-like protease (PL^pro^) from SARS-CoV-2, which contributed to drug-dependent suppression of viral replication.^7^ Here, we explored another putative mechanism of the antiviral activity of acriflavine, *i.e*., the inhibition of the interaction between the viral RBD domain of Spike protein and ACE2, the host receptor driving viral cell entry. Interaction between RBD and ACE2 in the presence of a vehicle (DMSO) was set to 100%. RBD exposed to increasing concentrations of acriflavine displayed diminished ACE2 binding capacity (Figure 6A). Upon preincubation with 156 nM of acriflavine, the unpaired mean difference in RBD:ACE2 interaction strength between vehicle and drug-exposed samples reached −17.5% [95.0%CI −27.3, −10.1]. The difference peaked at the highest assessed dose of 20 μM and was equal to −39.4% [95.0%CI −44.4, −34.7]. A dose–effect relationship between acriflavine concentration and RDB:ACE2 interaction followed the shape of a biphasic sigmoidal curve (Figure 6B). The first inhibitory tone occurred with IC50_1_ = 101 nM and plateaued at about −16% of basal interaction, whereas the second inhibitory tone was characterized by IC50_2_ = 7.3 μM and plateaued at −42% of basal interaction.

The biphasic characteristics of the observed dose–response curve may be related to the fact that acriflavine is a mixture of trypaflavine and proflavine, and that these compounds may display different inhibitory capacities. Another explanation involves the existence of two distinct binding sites at the surface of RBD.

The comparison of the dose–effect relationship between ACF concentration and RDB:ACE2 and the corresponding IC50 values lead to the conclusion that even for low-loading ACF@MOF composites, *i.e*., ACF@MOF-808 and ACF@UiO-66, the effective inhibition effect is achieved (Figure S11). The release of ACF from ACF@MOF-808 and ACF@UiO-66 composites was *ca*. 2 and 3.5 μM, respectively, which is still an order of magnitude higher than the measured first inhibitory tone (IC50_1_ = 101 nM).

### 3.6 Both Forms of Acriflavine Interact within RBD of SARS-CoV-2 Spike Model and at the RBD/ACE2 Interface

**Figure 7:**
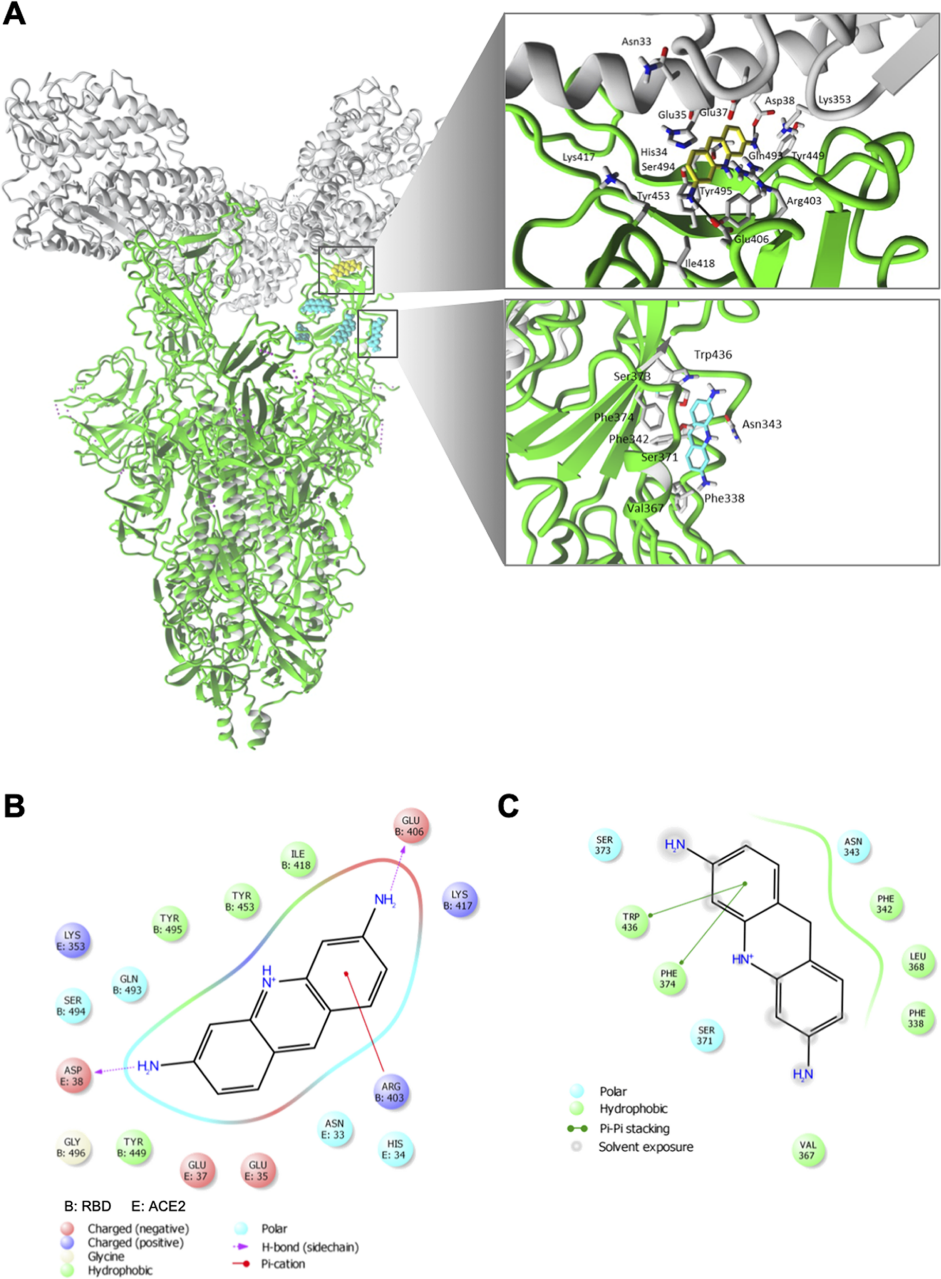
Molecular interactions of acriflavine (3,6-diaminoacridine) with RBD domain of human SARS-CoV-2 spike protein. (A) 3,6-Diaminoacridine binding sites (cyan; rendered in ball mode) are located within the RBD of SARS-CoV-2 trimer spike and at the interface between RBD and ACE2 (yellow; rendered in ball mode). More specifically, molecular docking results suggested four different sites of 3,6-diaminoacridine within RBD (all poses are shown in cyan) as presented in Table 2. The energetically lower poses within the RBD (cyan, rendered in stick mode) and at the interface between RBD and ACE2 (yellow, rendered in stick mode) were selected and presented with residues involved in binding (rendered in stick mode, element color code). Hydrogen bonds are marked with black arrows. All nonpolar hydrogen atoms are hidden. (B, C) 2D views of 3,6-diaminoacridine interacting at the interface between RBD and ACE2 (B) and within RBD of SARS-CoV-2 spike protein (C).

To determine the binding site locations and structural components for acriflavine at RBD of SARS-CoV-2 spike protein, we performed a molecular docking of 3,6-diaminoacridine and 3,6-diamino-10-methylacridinium to the molecular model of the human SARS-CoV-2 spike protein. Molecular docking results suggested similar binding sites for both 3,6-diaminoacridine (Figure 7 and Table S7) and 3,6-diamino-10-methylacridinium (Table S7 and Figure S21) at the RBD of SARS-CoV-2 model. More specifically, each molecule interacts with several possible binding sites within the RBD and binding site at the RBD/ACE2 interface (see Table S7). As it is presented in Figure 7A,B, 3,6-diaminoacridine interacts at the RBD/ACE2 interface mainly by cation−π interactions with Arg403 and hydrogen bonds formed between the carbonyl group of Glu406 and the amino group of the ligand as well as between carbonyl group of Asp38 and another amino group of the ligand. Other residues involved in binding at RBD/ACE2 interface are presented in Figure 7B and Table S7.

Molecular docking results suggested that 3,6-diaminoacridine, similarly to 3,6-diamino-10-methylacridinium, may bind to several sites within the RBD of the SARS-CoV-2 spike model. In the energetically lowest orientation of 3,6-diaminoacridine, located within the RBD, we observed π–π stacking with Trp436 and Phe374 as well (Figure 7A,C). Other residues involved in binding are included in Table S7.

Based on the docking results we suggest that there are several possible binding sites, and it may explain why we observed the biphasic characteristics of the dose–response curve presented in Figure 6.

It has been reported that hesperidin (anti-inflammatory, antioxidant natural compound) could target the binding interface between the SARS-CoV-2 spike and ACE2,^45^ and its mode of binding was used as an anti-SARS-CoV-2 drug screening strategy.^46^ This hesperidin binding site overlaps the suggested RBD/ACE2 interface site for both forms of acriflavine.

### 3.7 Acriflavine Displays Acceptable Safety Profile of Acriflavine-Bearing MOFs

**Figure 8:**
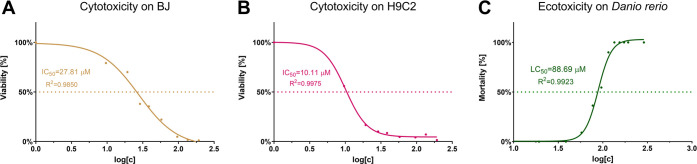
(A) Cytotoxicity of ACF performed on BJ and (B) H9C2. Viability (%) as a function of log[*c*] data was fitted to a biphasic dose–response curve. Calculated IC50s were 27.81 and 10.11 μM, respectively. (C) Ecotoxicity performed on *D. rerio* experimental model with the use of the FET test (OECD, test no. 236). Mortality (%) as a function of log[*c*] data was fitted to a biphasic dose–response curve. Calculated LC_50_ = 88.69 μM.

*In vitro* toxicity experiments were performed on epithelial cells derived from the skin (BJ cell line) and on cardiomyocytes (H9C2 cell line). In the first phase of experiments, a range of ACF concentrations was studied to establish IC50 for both cell lines. The values calculated directly from the curve log[*c*] *vs* viability(%) were equal to 27.81 and 10.11 μM, respectively (Figure 8). Then, based on the obtained values and release study, the effectiveness and safety profile of ACF@MOFs was studied in comparison to ACF.

*In vivo* toxicity studies performed using *D. rerio* demonstrated an acceptable safety profile of ACF@MOFs. In the initial fish embryo toxicity test (Figure 8C), ACF alone yielded the LC_50_ value equal to 88.69 μM. To evaluate the effectiveness and safety of MOFs used as an ACF carrier, similar experiments were performed, but the amounts of ACF and ACF@MOFs were set close to the lethal dose according to data from the release studies. The observation performed after 96 h proved that MOFs application as an ACF carrier improved the safety profile and decreased the mortality rate in comparison to ACF alone (Figure S23). Moreover, heart function studies (heartbeats per minute, Figure S24) also demonstrated that ACF released from MOFs was safer and less cardiotoxic in comparison to free ACF (see Supporting data).

## 4 Conclusions

This work aimed to comprehensively understand the structure–drug delivery–drug efficiency interactions in acriflavine (ACF) delivery systems based on zirconium-based metal–organic frameworks. In this work, the ACF was effectively loaded into the MOF-808, UiO-66, UiO-67, and NU-1000 metal–organic frameworks. The prepared ACF@MOF composites revealed that the loading efficiency is not only limited by the pore volume in each MOF structure but mainly by the presence of ions on the nodes of the metal–organic frameworks. Within the group of zirconium-based MOFs, MOF-808 and UiO-66 were determined as exhibiting a low ACF loading (4.14 and 5.55 wt %, respectively), whereas UiO-67 and NU-1000 were considered as high-ACF-loading composites (43.53 and 47.62 wt %, respectively).

The DFT calculation results have shown that the π–π stacking together with electrostatic interaction plays an important role in acriflavine adsorption and release from selected MOF structures. The modeled adsorption energies are in very good agreement with experimental release rates in water. The ACF release from prepared ACF@MOF composites was found to exhibit different kinetics and amount depending on the environment. The most efficient release was found in the water environment for all prepared composites, while for PBS (pH = 5.5) and SBF environments, decreased and prolonged release was observed. The most efficient ACF composites, *i.e*., ACF@UiO-67 and ACF@NU-1000, have shown extremely different release profiles in the SBF environment, showing almost 24% ACF release for ACF@UiO-67 and 2% for ACF@NU-1000 at the same time.

The activity of acriflavine determined by *in vitro* experiments has proved its superior inhibition activity for SARS-CoV-2. A dose–effect relationship between acriflavine concentration and RBD:ACE2 interaction allowed us to determine two IC50 values; the lower IC50 value was equal to 101 nM, whereas the higher value was equal to 7.3 μM. The comparison of determined acriflavine IC50 values with determined ACF release values confirms that even for low-ACF-loading composites, the required effective inhibition ACF concentrations can be achieved.

The *in vitro* and *in vivo* experiments have confirmed the low cytotoxicity of both metal–organic frameworks and ACF@MOF composites. Considering the low required effective ACF concentration to achieve the SARS-CoV-2 inhibition effect, the controlled long-term release from prepared MOF composites allows for achieving the required effective ACF concentration while avoiding the ACF side effects. Additionally, in the *in vivo* experiment with the most cardiotoxic dose (30 μg/mL, 115.5 μM), we have proved the cardioprotective effect, expressed as improvement in heartbeats, of all MOFs applied to release ACF.

The results of this study show that MOFs constitute attractive carriers for acriflavine—a drug displaying promising potential for COVID-19 management. The synergic effect between low required effective concentration and long-term dosage from studied carriers makes ACF@MOF composites good candidates for clinical trials.

## Supporting Information Available

Synthetic procedures; structural characterization of prepared materials; PXRD analysis; N_2_ adsorption isotherms; SEM images; ^1^H NMR spectral analysis; ACF release profiles; periodic density functional theory (DFT) calculations; molecular docking simulations; and *in vivo* experiments (PDF)
Optimized structures (.cif files) (ZIP)

## Notes

For the experiment with larvae up to 5-dpf, agreement of the Local Ethical Commission is not required.
