# Supplementary material for: Zirconium-Based Metal–Organic Frameworks as Acriflavine Cargos in the Battle against Coronaviruses—A Theoretical and Experimental Approach
Source: ACS Appl Mater Interfaces. 2022 Jun 14;14(25):28615–27. doi: 10.1021/acsami.2c06420 (PMC9212192; doi:10.1021/acsami.2c06420)
Supplement: Supplementary file 1 — am2c06420_si_001.pdf [file am2c06420_si_001.pdf]

Supporting information

Zirconium-based metal-organic frameworks as acriflavine cargos in battle against coronaviruses - theoretical and experimental approach

Przemysław J. Jodłowski[1,], Klaudia Dymek[1], Grzegorz Kurowski[1], Jolanta Jaśkowska[1], Wojciech Bury[2,*], Marzena Pander[2], Sylwia Wnorowska[3], Katarzyna Targowska-Duda[4], Witold Piskorz[5], Artur Wnorowski[4], Anna Boguszewska-Czubara[3]*

[1] Faculty of Chemical Engineering and Technology, Cracow University of Technology, 24 Warszawska, 31-155 Kraków, Poland

[2] Faculty of Chemistry, University of Wrocław, 14 F. Joliot-Curie, 50-383 Wrocław, Poland

[3] Department of Medical Chemistry, 4A Chodzki, Medical University of Lublin, 20-093 Lublin, Poland

[4] Department of Biopharmacy, Medical University of Lublin, 4A Chodzki, 20-093 Lublin, Poland

[5] Faculty of Chemistry, Jagiellonian University, Gronostajowa 2, 30-387 Kraków, Poland

Corresponding authors emails: przemyslaw.jodlowski@pk.edu.pl, wojciech.bury@uwr.edu.pl

S1. Materials

Zirconium oxide dichloride octahydrate ($ZrOCl_2 \times 8H_2O$, Alfa Aesar, 98%), Zirconium chloride ($ZrCl_4$, Merck, 98%), 1,3,5-benzenetricarboxylic acid (H_3BTC, TCI, 98%), 1,1'-biphenyl-4,4'-dicarboxylic acid (BPDC, Angene, 95%), benzoic acid (Acros, 99%), formic acid (Chempur, 85%), hydrochloric acid (Eurochem, 35-38%), trifluoroacetic acid (TCI, 99%), *N,N*-dimethylformamide (karpinex, 98%), acetone (Chempur, 98%), deuterated dimethylsulfoxide (DMSO-d_6, Deutero, 99.8 atom% D), deuterated sulfuric acid (D_2SO_4, Sigma-Aldrich, 96-98% solution in D_2O, 99.5 atom% D), acriflavine hydrochloride (Sigma-Aldrich) were used as received. 1,3,6,8-tetrakis(*p*-benzoic-acid)pyrene (H_4TBAPy) was synthesized following the literature protocol[1].

S2. Instrumentation

Powder XRD data were collected on Bruker D8 ADVANCE diffractometer equipped with a copper lamp (CuKα radiation, λ = 1.5406 Å) at 30 kV and 40 mA with a slit of 0.1°. Standard measurements were done in 2θ range of 4°-40° with a 2θ step of 0.008° and a counting time of 0.5 s.

Nitrogen sorption isotherms of Zr-MOF samples were measured at 77 K on a Micromeritics ASAP 2020 instrument. The samples were degassed at 120 °C for 24 h before the sorption measurement. The Brunauer-Emmett-Teller (BET) theory was used to calculate the specific surface areas of obtained materials. For all N_2 isotherm analyses we ensured that the two consistency criteria described by Roquerol et al.[8] and Walton et al.[9] were satisfied.

^1H nuclear magnetic resonance (NMR) spectra were recorded using a Bruker Avance 500 spectrometer at 298 K and were calibrated on the residual solvent signal (DSMO-d_6: 2.50 ppm).

ATR-FTIR spectra were collected by using the Thermo is10 spectrometer equipped with MCT detector. The spectra of pristine MOF samples as well as ACF@MOF composites was

measured by averaging 128 scans with 4 cm^{-1} resolution. ATR-FTIR measurements were performed without sample modification.

SEM images were collected on a Hitachi S-3400N-II variable-pressure scanning electron microscope. Samples were sputter-coated with 7 nm Au to facilitate viewing by SEM.

UV-Vis spectra were recorded by using AvaSpec-ULS3648 high-resolution spectrometer. The transmission cuvette holder CH-MP with signal enhancing mirrors CH-MP-EM equipped with optical fibers OF-UV-600-50-SS and an Ocean Optics DH-2000-BAL UV−vis−NIR light source was used during the experiments.

S3. Synthetic procedures

S3.1 Pristine MOF materials

MOF-808 material was obtained following the procedure of Dai *et al*[2]. Typically, 0.15 g (0.71 mmol) of 1,3,5-benzenetricarboxylic acid (H_3BTC) was dissolved in 12 ml of DMF, and then 0.22 g (0.67 mmol) of $ZrOCl_2 \times 8H_2O$ and 13 mL of formic acid were added to the mixture. The solution was stirred then at room temperature for 20 min and then heated at 110 °C for 2 days. The obtained solid was filtrated and resuspended in 30 ml of DMF, the mixture was then additionally heated at 100 °C overnight. The obtained material was washed with DMF (3 × 20 mL), followed by acetone (3 × 20 mL) and dried at 80 °C overnight.

UiO-66 material was synthesized according to the procedure described by Jodłowski *et al*.[3] following the procedure for the synthesis of defective UiO-66 50% HCl.

UiO-67 material was synthesized following the procedure described by Gutterød *et al*.[4] with some minor modifications. Typically, a glass vial was loaded with 0.54 g (2.32 mmol) of $ZrCl_4$, 9 ml of DMF and 250 μL of distilled water. The prepared milky solution was stirred at 80 °C for 10 min and 0.85 g (6.95 mmol) of benzoic acid was added to the mixture. The mixture was additionally sonicated for 10 min to ensure complete dissolution of benzoic acid and then 0.56 g

(2.32 mmol) of 1,1'-biphenyl-4,4'-dicarboxylic acid (BPDC) was added to the solution. The reaction mixture was heated and stirred at 130 °C for 16 h. The resulting solid was filtered and washed several times with DMF (5 × 20 mL). The obtained product was then washed with acetone (3 × 20 mL) and dried overnight at 80 °C.

Synthesis of NU-1000 material was performed following the procedure described by Sha et al.[5] at the scale of 0.4 g of the H_4TBAPy ligand.

Prior to the preparation of acriflavine@MOF (MOF-808, UiO-66, UiO-67, NU-1000) composites, the samples were activated in a Schlenk tubes under vacuum at 120 °C for 24 h.

S3.2 ACF@MOF composites

The acriflavine@MOF (MOF-808, UiO-66, UiO-67, NU-1000) composites were prepared according to the procedure described by Jodłowski *et al.*[3] with some modifications. Typically, 100 mg of acriflavine hydrochloride was dissolved in 10 mL of deionized water and subsequently mixed with 100 mg of previously activated metal organic framework for 24 h. Subsequently, as received acriflavine@MOF composites were centrifuged at 6000 rpm for 10 min, washed with 10 ml of deionised water. The as received suspension was centrifuged at 6000 rpm for 10 min and dried under ambient conditions overnight. The resulting acriflavine@MOF composites were denoted as: ACF@MOF-808, ACF@UiO-66, ACF@UiO-67, and ACF@NU-1000.

The amount of acriflavine loading in prepared ACF@MOF composites was determined by UV-Vis spectroscopy, by soaking the composites in deionised water for 24 h, followed by ultrasonic MOF disintegration of ultrasonic bath for 1 h. The resulting mixture was subsequently centrifuged at 6000 rpm for 10 min and the acriflavine concentration was determined by UV-Vis spectroscopy. The ACF loadings were calculated using the following formula:

$$\mathrm{ACF\ (wt\%)} = \frac{\mathrm{m_{ACF}}}{m_{\mathrm{ACF}} + \mathrm{m_{MOF}}} \cdot 100$$

where:

$\mathbf{m_{ACF}}$ - the determined mass of ACF in measured solution (mg),

$\mathbf{m_{MOF}}$ – the mass of MOF samples used during ultrasonic MOF disintegration (mg).

S4. Structural characterization of prepared materials

S4.1 PXRD analysis

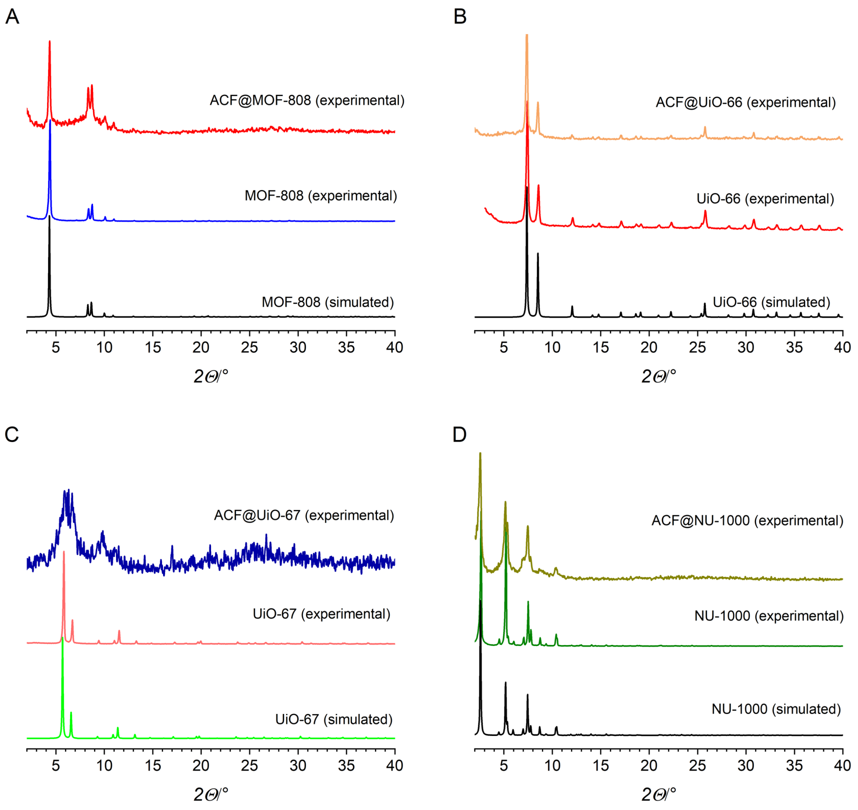

Figure S1. Powder X-ray diffraction patterns for pristine MOF samples and ACF@MOF composites: (**A**) MOF-808 and ACF@MOF-808, (**B**) UiO-66 and ACF@UiO-66, (**C**) UiO-67 and ACF@UiO-67, (**D**) NU-1000 and ACF@ NU-1000

S4.2 N_2 adsorption isotherms

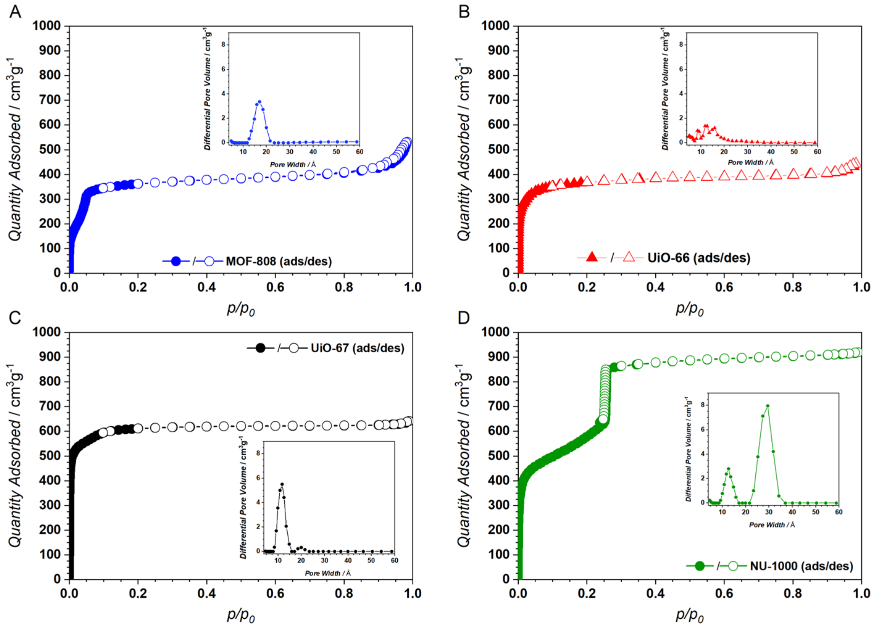

Figure S2. Nitrogen sorption isotherms measured at 77 K for pristine Zr-MOFs: (**A**) MOF-808, (**B**) UiO-66, (**C**) UiO-67, (**D**) NU-1000; insets - density functional theory (DFT) pore size distribution (PSD) for pristine Zr-MOFs

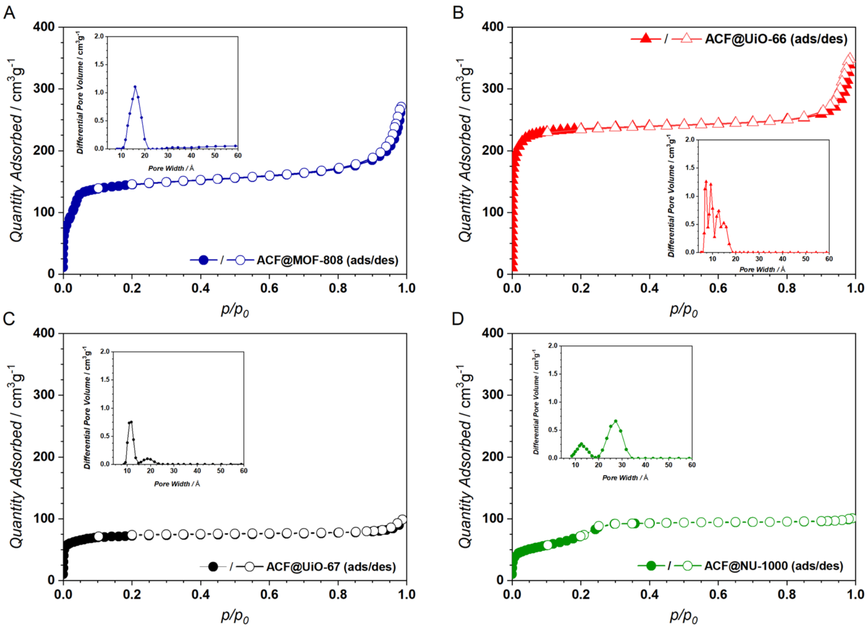

Figure S3 Nitrogen sorption isotherms measured at 77 K for ACF@MOF composites: (**A**) ACF@MOF-808, (**B**) ACF@UiO-66, (**C**) ACF@UiO-67, (**D**) ACF@NU-1000; insets- density functional theory (DFT) pore size distribution (PSD) ACF@MOF composites

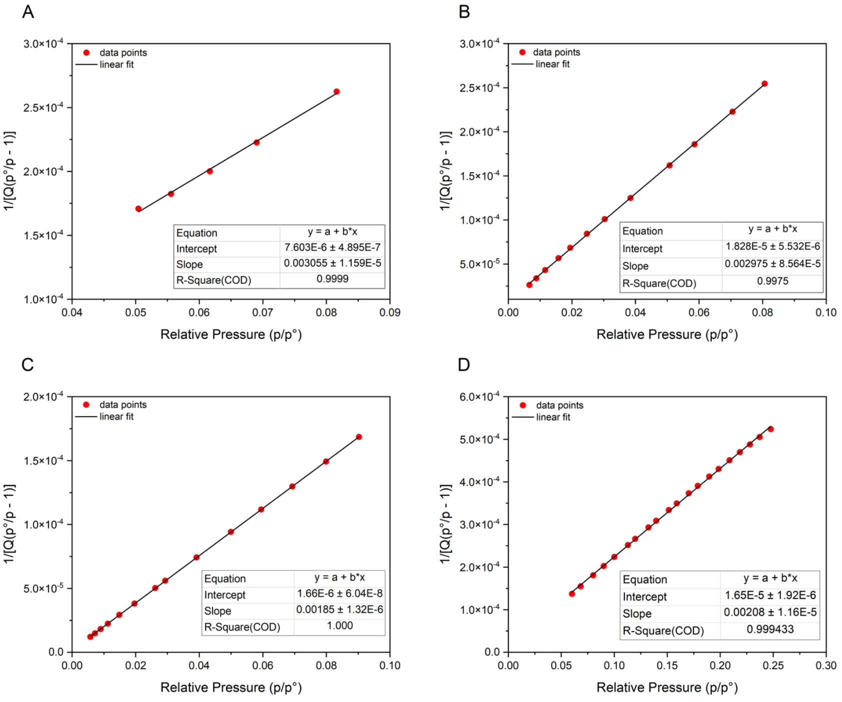

Figure S4. BET plots and fitting parameters for pristine Zr-MOFs: (**A**) MOF-808, (**B**) UiO-66, (**C**) UiO-67, (**D**) NU-1000.

S4.3. SEM images

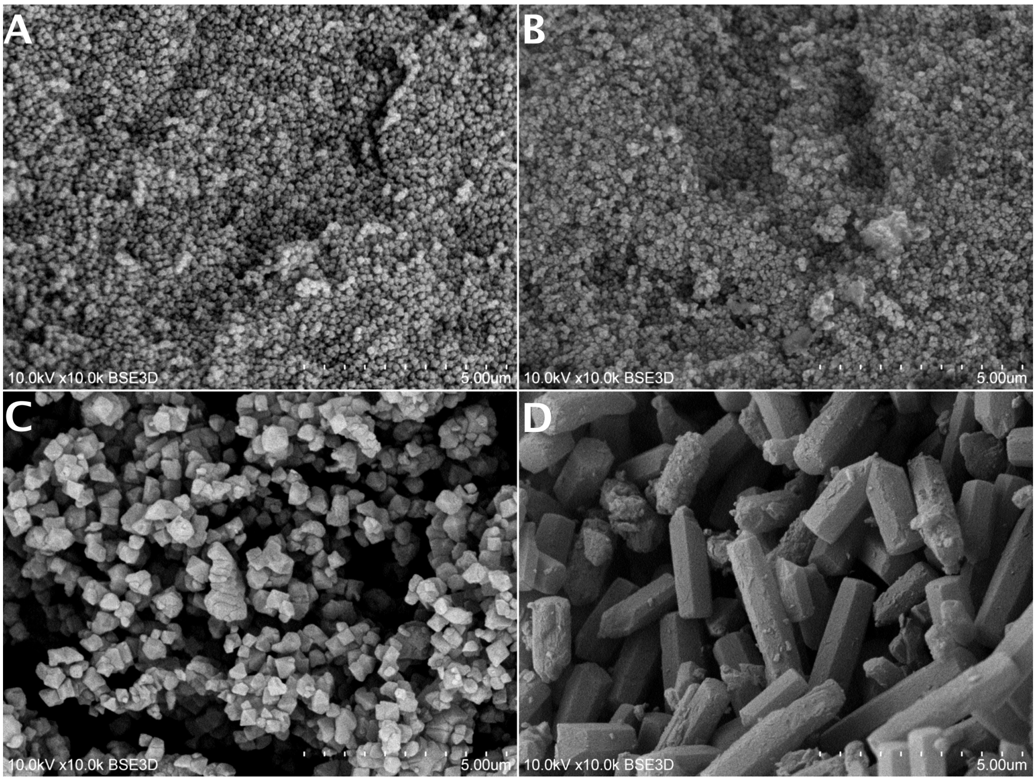

Figure S5. SEM images of pristine Zr-MOFs: (**A**) MOF-808, (**B**) UiO-66, (**C**) UiO-67, (**D**) NU-1000.

S4.4. ^{1}H NMR spectral analysis

The NMR samples of obtained MOFs before and after incorporation of acriflavine were prepared by digesting approximately 2 mg of the dried material in D_2SO_4 solution and then diluting it with 0.6 ml of DMSO-d_6.

The number of incorporated acriflavine molecules in the MOF structures n was calculated directly from measured ^{1}H NMR spectra of obtained ACF@MOF samples (Figures S6-S9). The selected set of singlets at 8.7 ppm were assigned to different forms of acriflavine (1 H) and was integrated against proton signals of the corresponding MOF linkers. The ^{1}H NMR spectrum of acriflavine in DMSO-d_6 was used as a reference (Figure S5).

The weight percentage of the introduced acriflavine molecule (ACF) in the whole material was calculated as follows:

$$\text{ACF (wt\%)} = \frac{n \cdot \mathrm{M_{ACF}}}{n \cdot \mathrm{M_{ACF}} + \mathrm{M_{MOF}}} \cdot 100$$

where:

$\boldsymbol{n}$ is the number of the IM per Zr-node (equal the number of IM per 1 mol of respective MOF),

$\mathbf{M_{ACF}}$ - the molecular mass of the introduced molecule (259.74 g/mol),

$\mathbf{M_{MOF}}$ - the molecular mass of respective MOF related to one Zr_6 node.

Table S1. Results of ACF loadings for ACF@MOF composites based on ^{1}H NMR data.

**Zr-MOF**			**ACF**	
**MOF**	**Chemical formula**	**Molecular mass**	**n[1]**	**wt%**
MOF-808	$Zr_6O_4(OH)_{10}(H_2O)_6(C_9H_3O_6)_2$	1303.7 g/mol	0.06	1.2
UiO-66	$Zr_6O_4(OH)_4(C_8H_4O_4)_6$	1664.1 g/mol	<0.01	<0.5
UiO-67	$Zr_6O_4(OH)_4(C_{14}H_8O_4)_6$	2120.7 g/mol	4.7	36
NU-1000	$Zr_6O_4(OH)_8(H_2O)_4(C_{44}H_{22}O_8)_2$	2176.8 g/mol	4.9	37

[1] based on analysis of corresponding NMR spectra of **ACF@MOF** samples

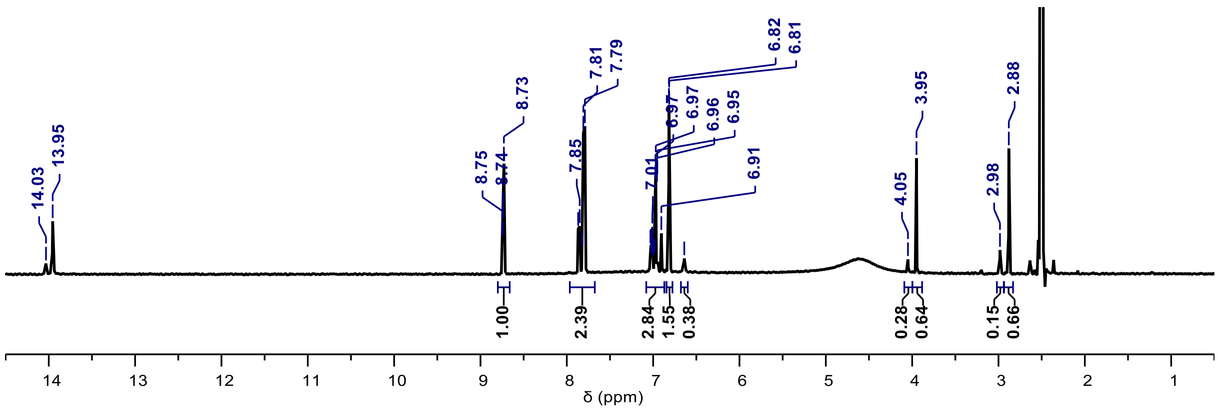

Figure S6. ^{1}H NMR spectrum of commercial acriflavine in DMSO-d_6.

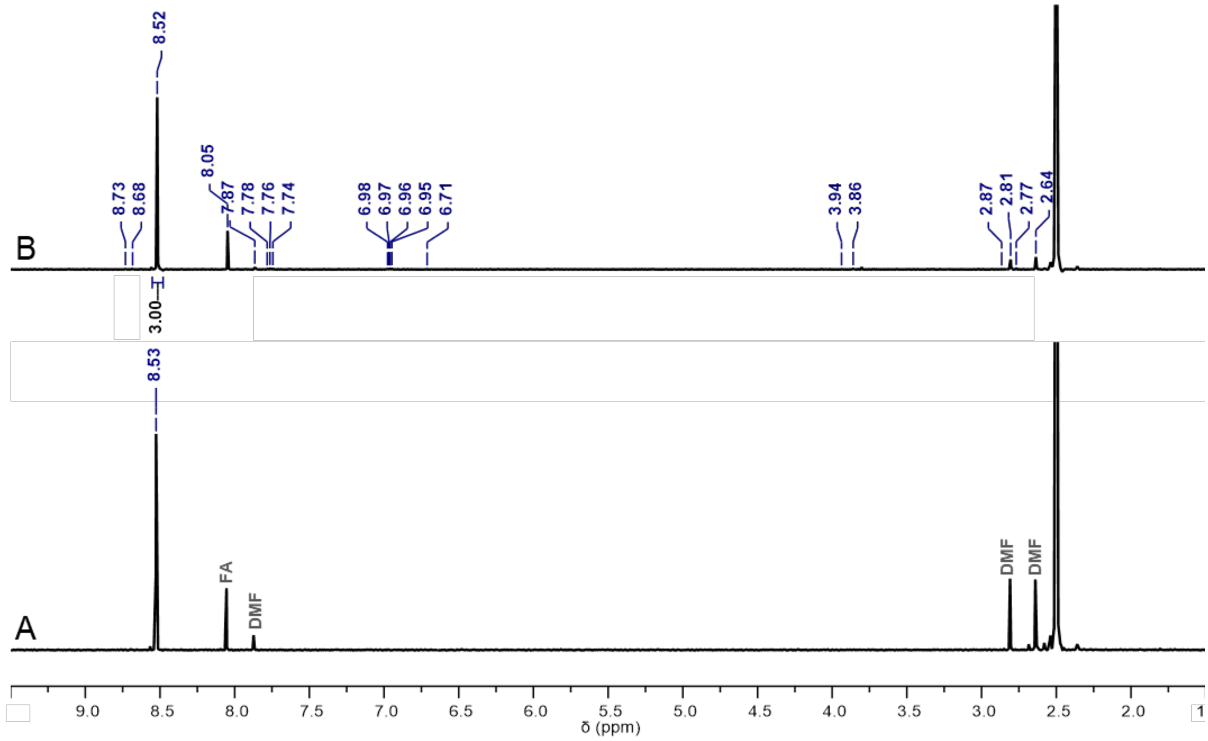

Figure S7. ^{1}H NMR spectra of the **MOF-808** material digested in D_2SO_4/DMSO-d_6 solution before (**A**) and after (**B**) incorporation of acriflavine.

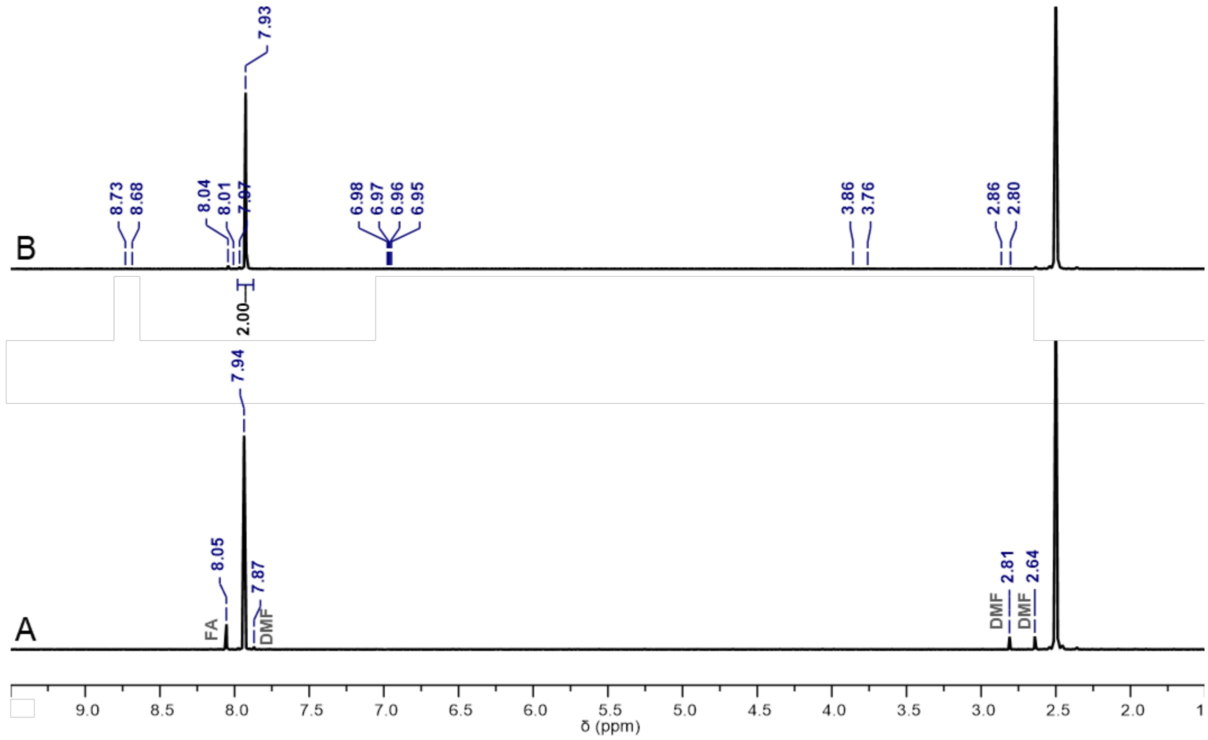

Figure S8. ¹H NMR spectra od the **UiO-66** material digested in D_2SO_4/DMSO-d_6 solution before (**A**) and after (**B**) incorporation of acriflavine.

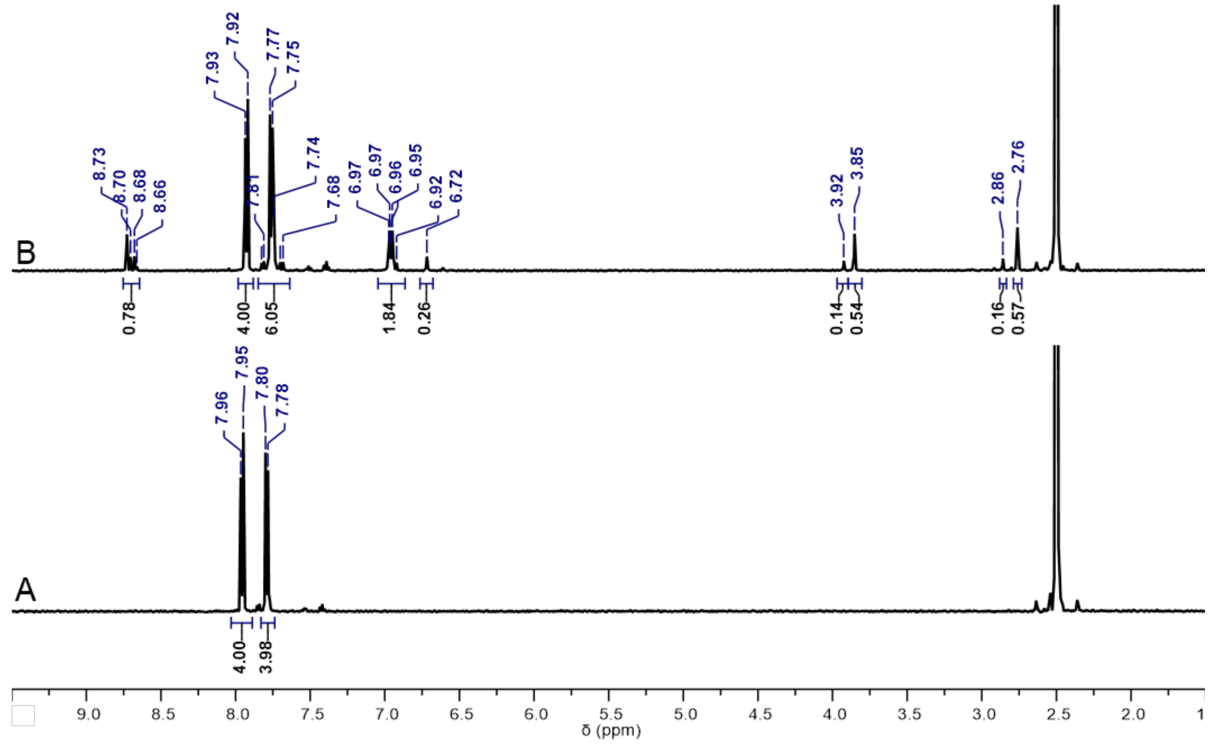

Figure S9. ¹H NMR spectra of the **UiO-67** material digested in D_2SO_4/DMSO-d_6 solution before (**A**) and after (**B**) incorporation of acriflavine.

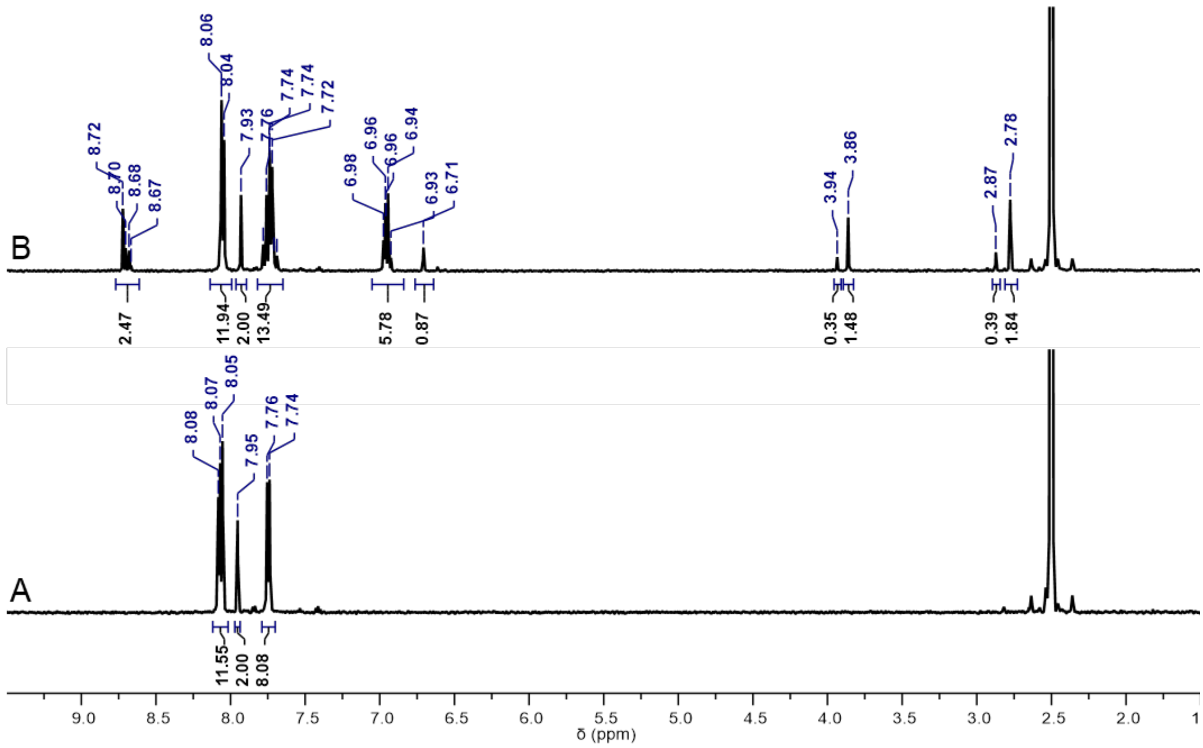

Figure S10. ^{1}H NMR spectra of the NU-1000 material digested in D_2SO_4/DMSO-d_6 solution before (**A**) and after (**B**) incorporation of acriflavine.

S4.5 ACF release profiles

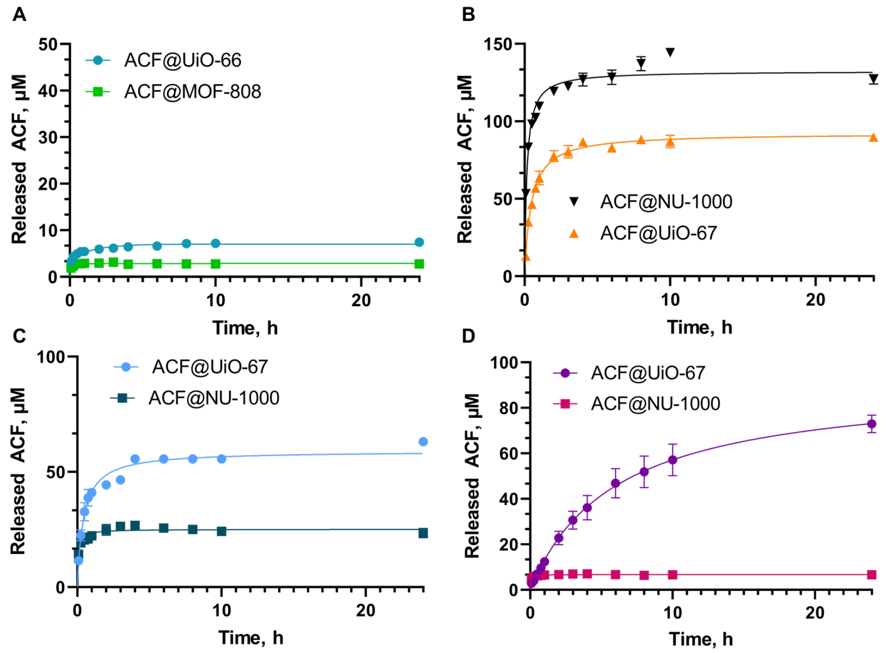

Figure S11. Acriflavine (ACF) release profiles for ACF@MOF composites: (**A**), (**B**) in deionized water, (**C**) in PBS pH = 5.5, (**D**) in SBF.

S5. Periodic density functional theory (DFT) calculations

The periodic density functional theory (DFT) calculations were performed with the use of the VASP[6,7] code utilizing the PAW[8,9] method to reconstruct all–electron wave function. The PBE[10] correlation-exchange functional was used and, to account for the van der Waals dispersion interactions, the semi-empirical method parametrized by Grimme[11,12] was applied. The plane wave basis set energy cut-off was chosen as 450 eV. As a convergence accelerator, the Gaussian smearing of the Fermi-Dirac distribution was used with energy width of 0.01 eV.

Due to the large size of the unit cells (see below), the Γ-only *k*-space sampling was found to be sufficient allowing for the separation of the *k*-points in the Irreducible Brillouin Zone.

To compare the adsorption energies among different MOFs, aware of the incomplete basis set, in case of each MOF structure the adsorption energy was calculated with use of the total energy of a single acriflavine molecule (separately 3,6-diaminoacridine or 3,6-diamino-10-methylacridine) in the unit cell of the given MOF. Unit cell parameters of the computational models are provided in Table S2.

The energetics of sorption is given in Table S3 and the geometric relations are presented in Table S4. As can be seen, for NU-1000 the structure 2 did not exhibit a π-π stacking (orbital interaction) and the sorption energy was lower (to the absolute value) by *ca.* 0.2 eV with respect to the structure 1.

Table S2. Unit cell parameters of the computational models.

MOF	Stoichiometry	Unit cell vectors length / Å			Unit cell vectors angles / °		
		h	*k*	*l*	α	β	γ
**MOF-808**[13]	$C_{96}H_{64}Zr_{24}O_{128}$	25.001	25.000	24.997	59.70	59.72	59.72
**UiO-66**[14]	$C_{192}H_{96}Zr_{24}O_{128}$	20.747	20.747	20.747	90.0	90.0	90.0
**UiO-67**[14]	$C_{84}H_{52}Zr_{6}O_{32}$	19.012	19.012	19.012	60.0	60.0	60.0
**NU-1000**[15]	$C_{264}H_{180}Zr_{18}O_{96}$	39.970	40.000	16.580	90.0	90.0	120.0

Table S3. Energetics of 3,6-diamino-10-methylacridine and 3,6-diaminoacridine sorption in studied MOF structures. The calculated loading $n_{molecules}$ was estimated per 1 unit cell of MOF.

Adsorbate	E_{ads} (vac.) / eV	E_{ads} (water)* / eV	$n_{molecules}$** calculated	$n_{molecules}$*** experimental
**MOF-808**				
3,6-diamino-10-methylacridine	-0.752	-0.636	13 (3.25)	0.91
3,6-diaminoacridine	-0.648	-0.539	18 (4.5)	1.12
3,6-diaminoacridine hydrochloride	-0.772	-0.524	18 (4.5)	0.96
**UiO-66**				
3,6-diamino-10-methylacridine	-1.865	-2.749	0 (0)	1.50
3,6-diaminoacridine, struct. 1	-1.958	-2.698	0 (0)	1.87
3,6-diaminoacridine, struct. 2	-1.986	-2.691		
3,6-diaminoacridine hydrochloride, struct. 1	-1.433	-1.960	0 (0)	1.59
**UiO-67**				
3,6-diamino-10-methylacridine, struct. 1	-1.032	-0.697	5 (5)	6.30
3,6-diamino-10-methylacridine, struct. 2	-1.163	-0.537		
3,6-diaminoacridine, struct. 1	-0.716	-0.619	6 (6)	7.81
3,6-diaminoacridine, struct. 2	-0.835	-0.744		
3,6-diaminoacridine hydrochloride, struct. 1	0.274	-0.689	6 (6)	6.65
**NU-1000**				
3,6-diamino-10-methylacridine, struct. 1	-0.987	-0.263	29 (9.67)	22.86
3,6-diamino-10-methylacridine, struct. 2	-0.636	-0.402		
3,6-diaminoacridine, struct. 1	-0.718	-0.627	39 (13.33)	28.38

3,6-diaminoacridine, struct. 2	-0.454	-0.464	39 (13.33)	24.17
3,6-diaminoacridine hydrochloride, struct. 1	-0.202	-0.672		

* Calculations performed with polarizable continuum (DFTsol) with ε = 80

** calculated within the rigid host approximation. Values in parentheses are per a single Zr_6 cluster.

*** values calculated from the experimental %wt measured for the mixture of 3,6-diaminoacridine and 3,6-diamino-10-methylacridine

Table S4. Geometrical details for the π-π stacking in acriflavine interaction with NU-1000, UiO-66, UiO-67 and MOF-808. The angle α is a dihedral between planes of the rings. Definition of distances and β, γ angles are given in Fig. S11.

Adsorbates	d(c(M)-c(L) / Å	d⊥(c(L)-P(M) / Å	d⊥(c(M)-P(L) / Å	a / °	b / °	g / °
**MOF-808**						
3,6-diamino-10-methylacridine	3.830	3.738	3.413	16.88	26.97	12.57
3,6-diaminoacridine	4.168	3.058	3.984	25.72	42.81	17.10
3,6-diaminoacridine hydrochloride	4.234	2.654	4.163	40.79	51.18	10.52
**UiO-66**						
3,6-diamino-10-methylacridine	3.097	3.094	3.094	0.53	2.90	2.61
3,6-diaminoacridine	2.044	2.024	2.039	4.58	8.04	3.93
3,6-diaminoacridine hydrochloride	3.163	3.144	3.143	2.43	6.30	6.43
**UiO-67**						
3,6-diamino-10-methylacridine, struct. 1	4.010	3.849	3.586	26.69	16.30	26.61
3,6-diamino-10-methylacridine, struct. 2	3.889	3.444	3.693	9.94	27.66	18.26
3,6-diaminoacridine, struct. 1	3.183	2.668	2.502	16.33	33.04	38.18
3,6-diaminoacridine, struct. 2	6.294	4.806	6.207	39.68	40.21	9.50
3,6-diaminoacridine hydrochloride, struct. 1	3.360	3.292	3.333	4.58	11.54	7.26
**NU-1000**						

3,6-diamino-10-methylacridine, struct. 1	2.887	2.847	2.868	3.145	9.51	6.44
3,6-diamino-10-methylacridine, struct. 2	no π–π stacking*					
3,6-diaminoacridine, struct. 1	3.598	3.550	3.565	1.865	9.39	7.81
3,6-diaminoacridine, struct. 2	no π–π stacking*					
3,6-diaminoacridine hydrochloride, struct. 1	3.581	3.504	3.475	3.536	11.97	14.00

c(*x*) – center of "*x*" ring

P(*x*) – plane fitted to the aromatic ring "*x*"

M – aromatic ring of adsorbed molecule

L – aromatic ring of the MOF linker

$d_{\perp}(x\text{-}y)$ – distance (in the normal direction) between *x* and *y*

* – optimized geometry does not allow for the π–π stacking due to the nil overlap imposed by the relative orientation of the aromatic platforms of M and L

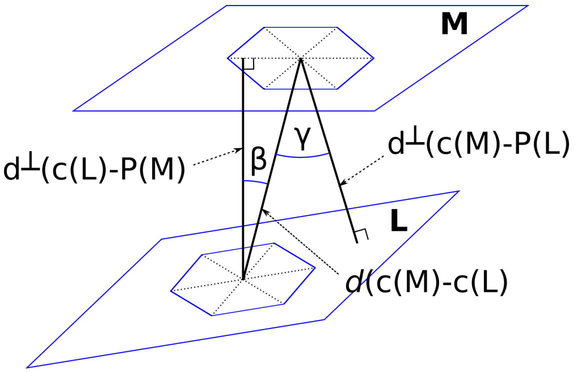

Figure S12. Sketch presenting the definition of the angles and distances whose values are shown in Table S3. The letters "M" and "L" denote the average planes of the aromatic rings of the adsorbed molecule and the linker, respectively.

Table S5. Calculated kinetic parameters of acriflavine release from NU-1000, UiO-66, UiO-67 and MOF-808. The equation numbering refers to the main text.

MOF	Drug release kinetics model	Model parameters
**MOF-808**/water	first order (eq. 1)	F_{max} = (2.860±0.021) μmol/dm^3<br>k_1 = (4.80±1.15) min^{-1}
**UiO-66**/water	first order (eq. 1)	F_{max} = (7.400±0.030) μmol/dm^3<br>k_1 = (0.448±0.017) min^{-1}
**UiO-67**/water	GC (eq. 2)	F_{max} = (43.510±0.197) μmol/dm^3<br>k_1 = (0.920±0.026) min^{-1}<br>k_2 = (12.19±16.83) min^{-1}<br>$t_{2,max}$ = (-0.341±6.21) min
**NU-1000**/water	GC (eq. 2)	F_{max} = (67.662±17.900) μmol/dm^3<br>k_1 = (4.681±0.142) min^{-1}<br>k_2 = (0.511±0.048) min^{-1}<br>$t_{2,max}$ = (-0.020±0.951) min
**UiO-67**/PBS	GC (eq. 2)	F_{max} = (29.253±4.658) μmol/dm^3<br>k_1 = (0.469±0.018) min^{-1}<br>k_2 = (7.657±1.077) min^{-1}<br>$t_{2,max}$ = (0.162±0.365) min
**NU-1000**/PBS	GC (eq. 2)	F_{max} = (23.483±0.313) μmol/dm^3<br>k_1 = (1.281±0.111) min^{-1}<br>k_2 = (-0.712±0.338) min^{-1}<br>$t_{2,max}$ = (8.733±0.722) min
**UiO-66**/SBF	first order (eq. 1)	F_{max} = (68.315±0.646) μmol/dm^3<br>k_1 = (0.184±0.004) min^{-1}
**NU-1000**/SBF	GC (eq. 2)	F_{max} = (6.636±0.297) μmol/dm^3<br>k_1 = (0.470±0.674) min^{-1}<br>k_2 = (-0.658±1.367) min^{-1}<br>$t_{2,max}$ = (1.667±15.106) min

Table S6. Summary of the geometrical properties of the MOF frameworks (aperture and pore diameters) and the ACF molecules (diameters) determined by the Accessible Solvent Surface. The pore and channel diameters were calculated as the maximal diameters of spheres fitting in the pore/channel. The margin (offset) of 1.4 Å has been taken into account.

Geometry descriptor	value / Å
**MOF-808**	
Wide aperture diameter	14.7
Small aperture diameter	10.9
Pore diameter	17.1
**UiO-66**	
Aperture diameter	3.6
Pore diameter	7.9
**UiO-67**	
Aperture diameter	6.1
Pore diameter	12.2
**NU-1000**	
Wide aperture diameter	28.7
Small aperture diameter	6.7
**3,6-diaminoacridine**	
d_X*	14.0
d_Y	7.8
d_Z	3.2
**3,6-diamino-10-methylacridine**	
d_X	14.2
d_Y	8.5
d_Z	8.4
**3,6-diaminoacridine · HCl**	
d_X	14.1
d_Y	7.8
d_Z	6.3
* the molecules have been arranged so as to diagonalize the envelope ellipse; The X, Y, and Z labels denote the axes along which the diameter was measured.	

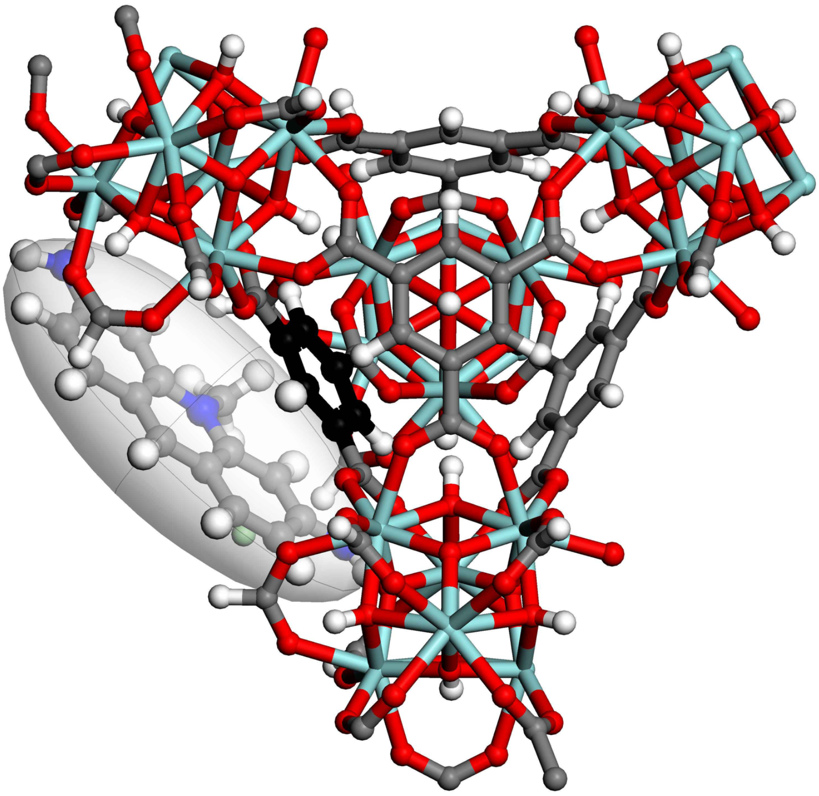

Figure S13. Optimized structure of 3,6-diamino-10-methylacridinium chloride@MOF-808.

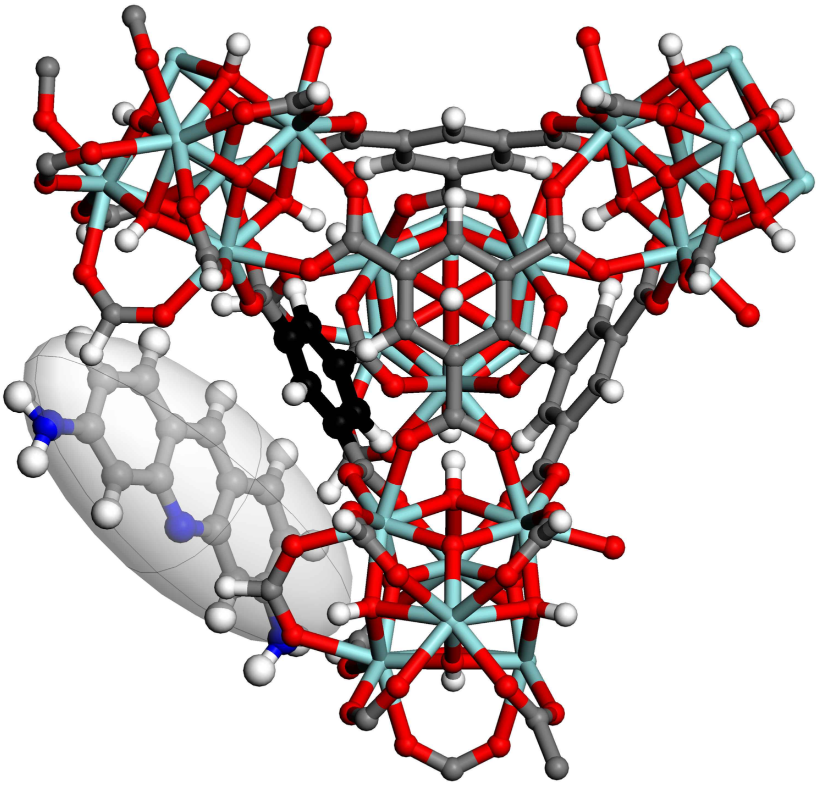

Figure S14. Optimized structure of 3,6-diaminoacridine@MOF-808.

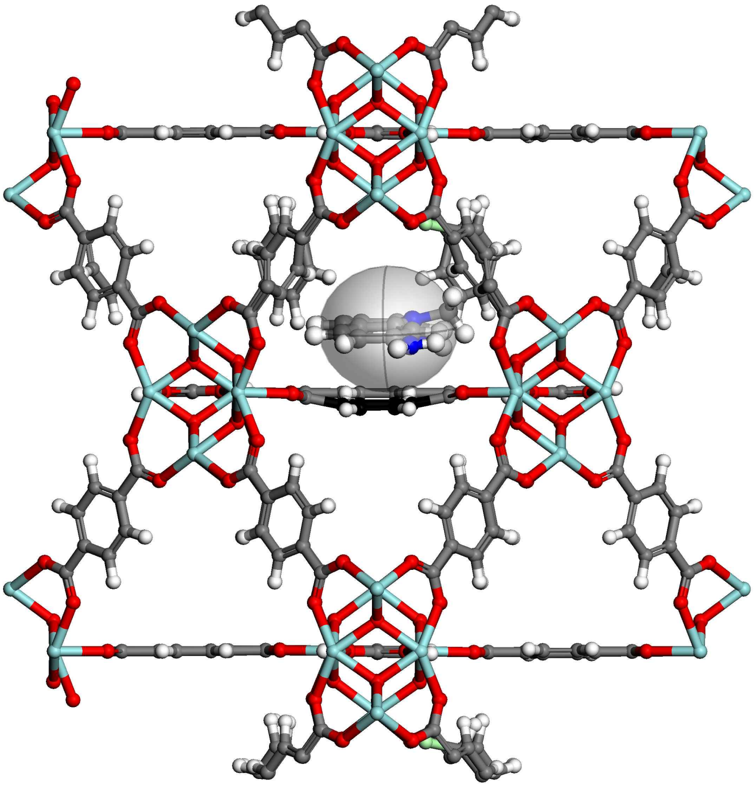

Figure S15. Optimized structure of 3,6-diamino-10-methylacridinium chloride@UiO-66.

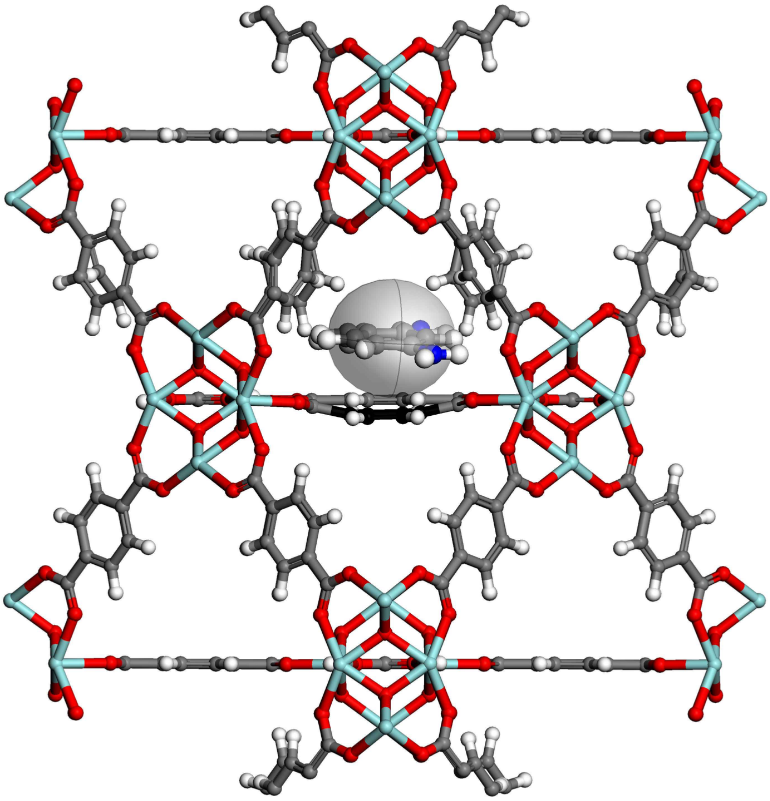

Figure S16. Optimized structure of 3,6-diaminoacridine@UiO-66.

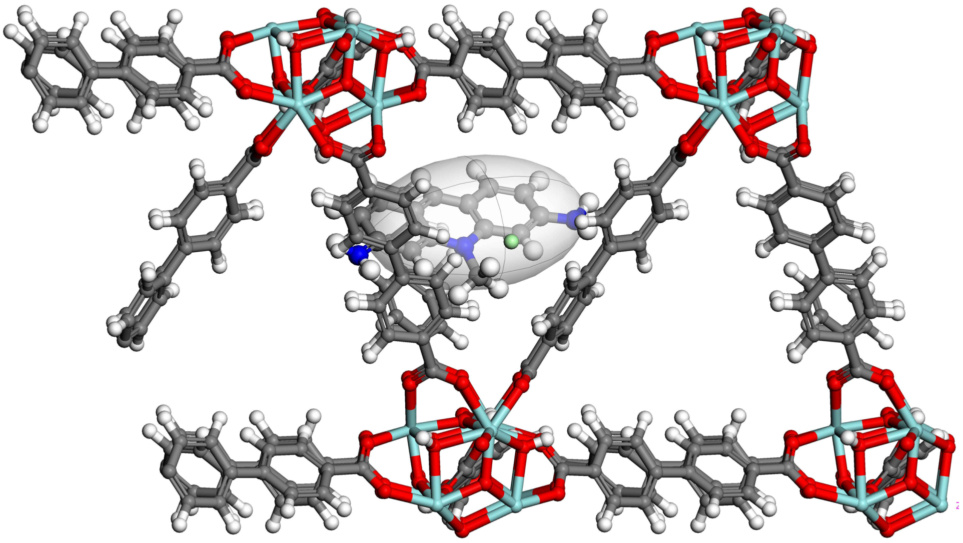

Figure S17. Optimized structure of (3,6-diamino-10-methylacridinium chloride)@UiO-67.

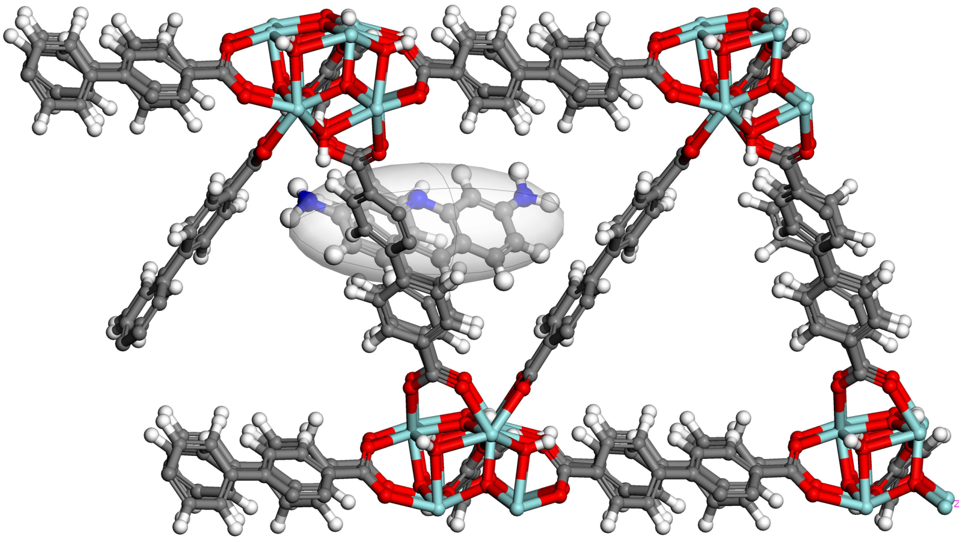

Figure S18. Optimized structure of 3,6-diaminoacridine@UiO-67.

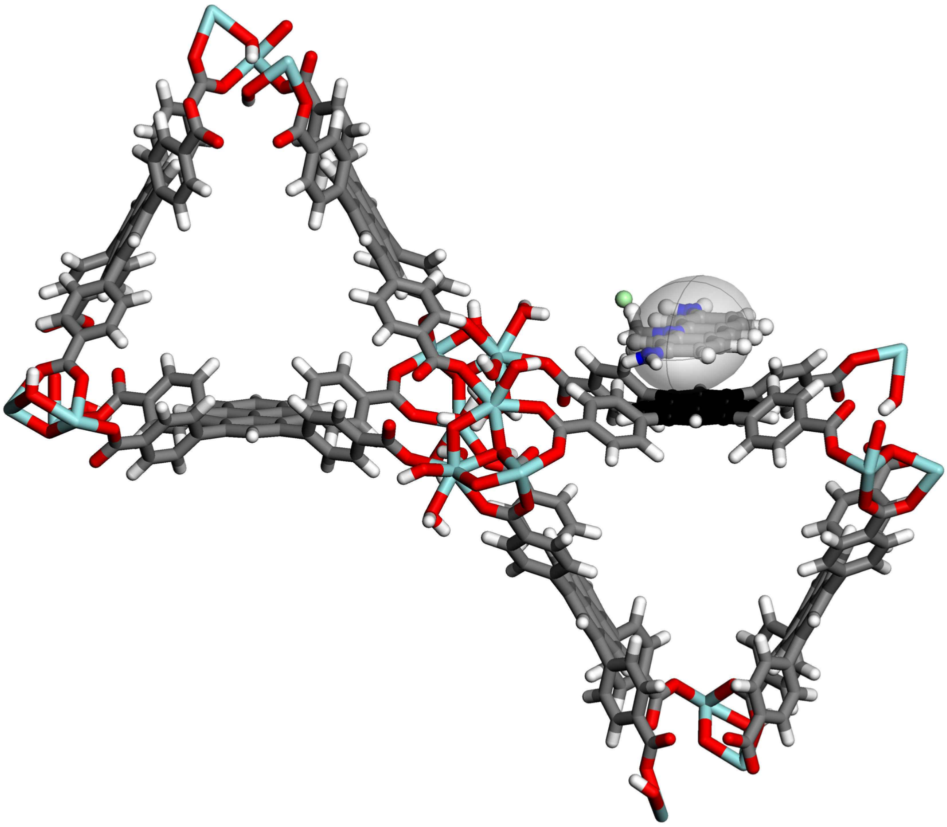

Figure S19. Optimized structure of (3,6-diamino-10-methylacridinium chloride)@NU-1000.

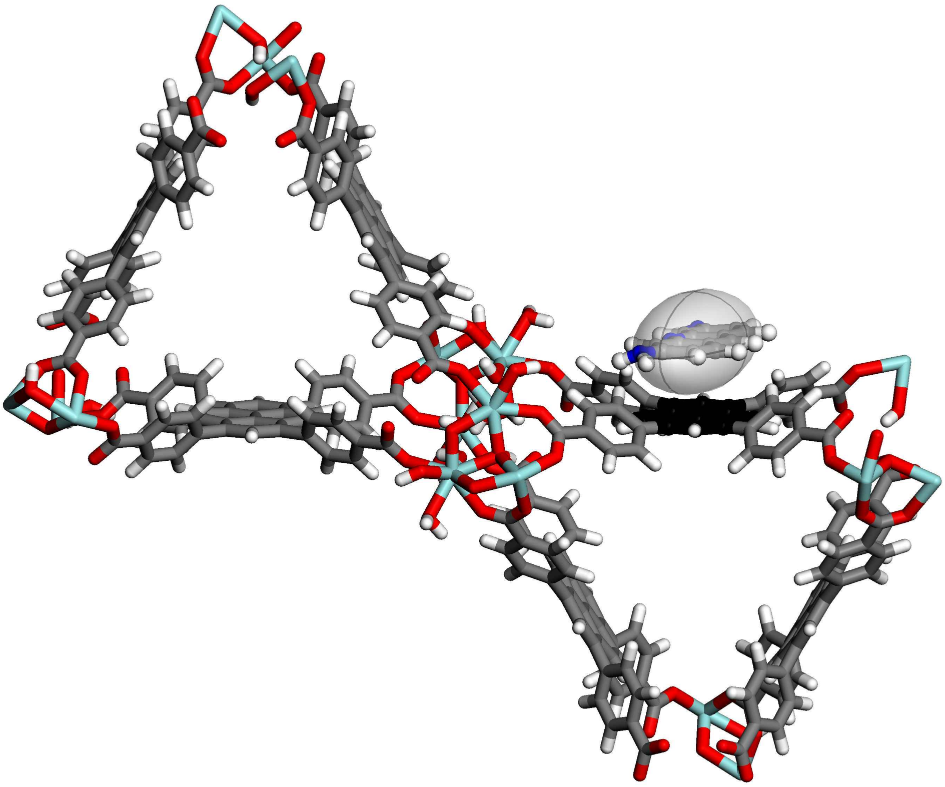

Figure S20. Optimized structure of 3,6-diaminoacridine@NU-1000.

S6. Molecular docking simulations

Table S7. Residues involved in 3,6-diaminoacridine and 3,6-diamino-10-methylacridinium binding to RBD and RBD/ACE2 interface.

Ligand	Binding site	Residues involved in binding	Calculated binding energy / (kcal/mol)
3,6-diaminoacridine	RBD/ACE2 interface	**ACE:**<br>Asn33<br>His34<br>Glu35<br>Glu37<br>Asp38<br>Lys353<br>**RDB:**<br>Arg403<br>Glu406<br>Lys417<br>Ile418<br>Tyr449<br>Tyr453<br>Gln493<br>Ser494<br>Tyr495<br>Gly496	-5.9
	Site 1 within RBD	Phe338<br>Phe342<br>Asn343<br>Leu368<br>Val367	-5.8

		Ser371<br>Ser373<br>Phe374	
	Site 2 within RBD	Tyr369<br>Phe374<br>Ser375<br>Thr376<br>Phe377<br>Pro384<br>Thr385	-5.6
	Site 3 within RBD	Pro426<br>Asp427<br>Asp428<br>Thr430<br>Lys462<br>Pro463<br>Phe464<br>Glu516	-5.1
	Site 4 within RBD	Thr376<br>Lys378<br>Tyr380<br>Agr408<br>Ala411<br>Pro412<br>Gly413<br>Gly414	-5.1
3,6-diamino-10-methylacridinium	Site 1 within RBD	Trp436<br>Phe338<br>Phe342	-5.9

		Asn343<br>Val 367<br>Leu368<br>Ser371<br>Ser373<br>Phe374	
	Site 2 within RBD	Tyr369<br>Phe374<br>Ser375<br>Thr376<br>Phe377<br>Pro384<br>Thr385	-5.7
	Site 3 within RBD	Pro426<br>Asp427<br>Asp428<br>Phe429<br>Thr430<br>Lys462<br>Pro463<br>Phe464<br>Glu516	-5.3
	Site 4 within RBD	Arg355<br>Tyr396<br>Phe464<br>Glu516<br>Thr167	-5.1
	RBD/ACE2 interface	**ACE:**<br>Asn33	-5.1

		His34<br>Glu35<br>Glu37<br>Asp38<br>Lys353<br>**RDB:**<br>Arg403<br>Glu406<br>Lys417<br>Ile418<br>Tyr453<br>Leu455<br>Gln493<br>Ser494<br>Tyr495<br>Gly496	

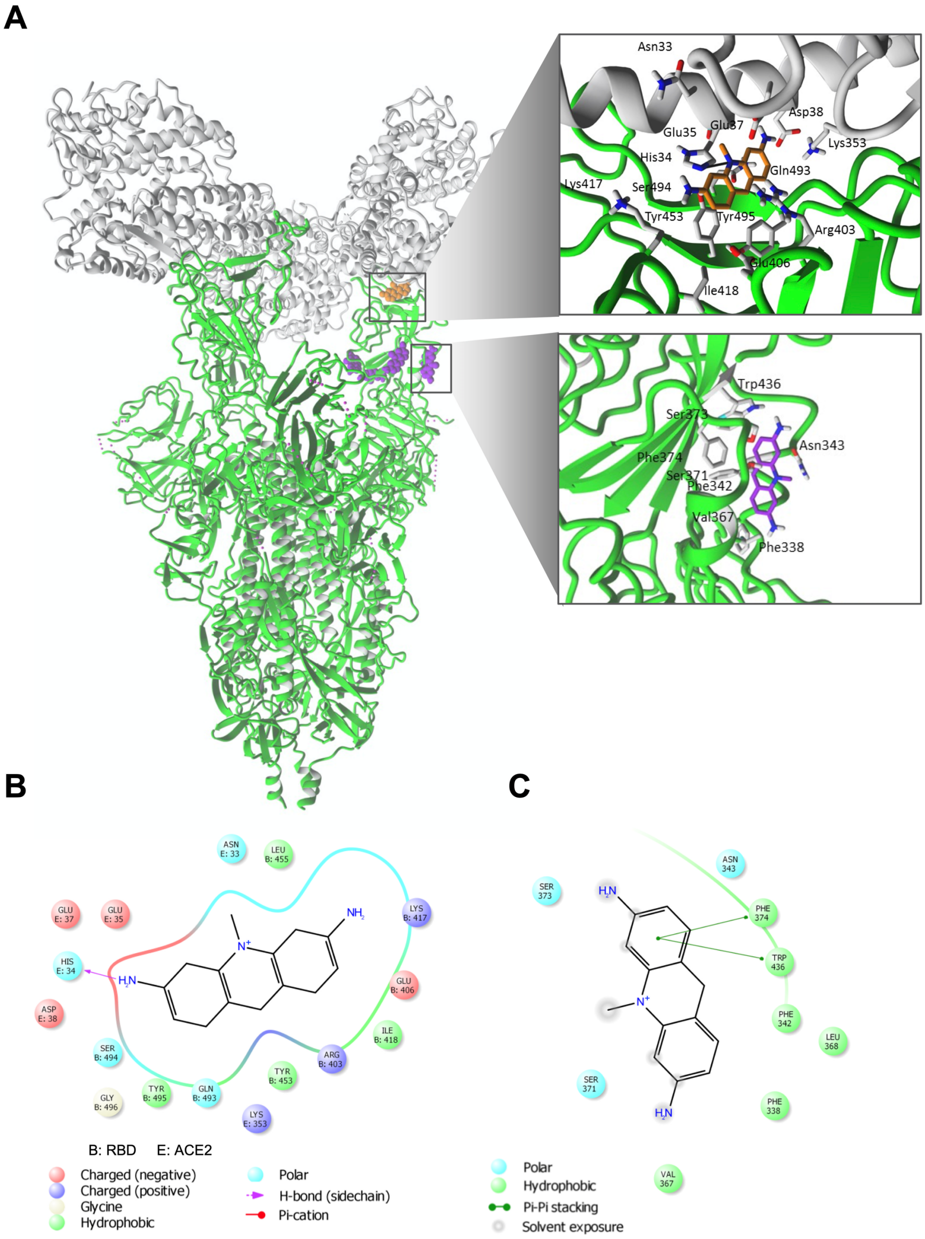

Figure S21. Molecular docking of 3,6-diamino-10-methylacridinium to human RBD of SARS-CoV-2 trimer spike. (**A**) 3,6-diamino-10-methylacridinium binding sites (purple; rendered in ball mode) are located within the RBD of SARS-CoV-2 trimer spike and at the interface between RBD and ACE2 (orange; rendered in ball mode). More specifically, molecular docking results suggested four different sites of 3,6-diamino-10-methylacridinium within RBD (all poses are shown in purple) as presented in Table 2. The energetically lower poses within the RBD (purple, rendered in stick mode) and at the interface between RBD and ACE2 (orange,

rendered in stick mode) were selected and presented with residues involved in binding (rendered in stick mode, element color code). All non-polar hydrogen atoms are hidden. (B-C) 2D views of 3,6-diamino-10-methylacridinium interacting at the interface between RBD and ACE2 (panel **B**) and within RBD (panel **C**).

S7. *In vivo* experiments

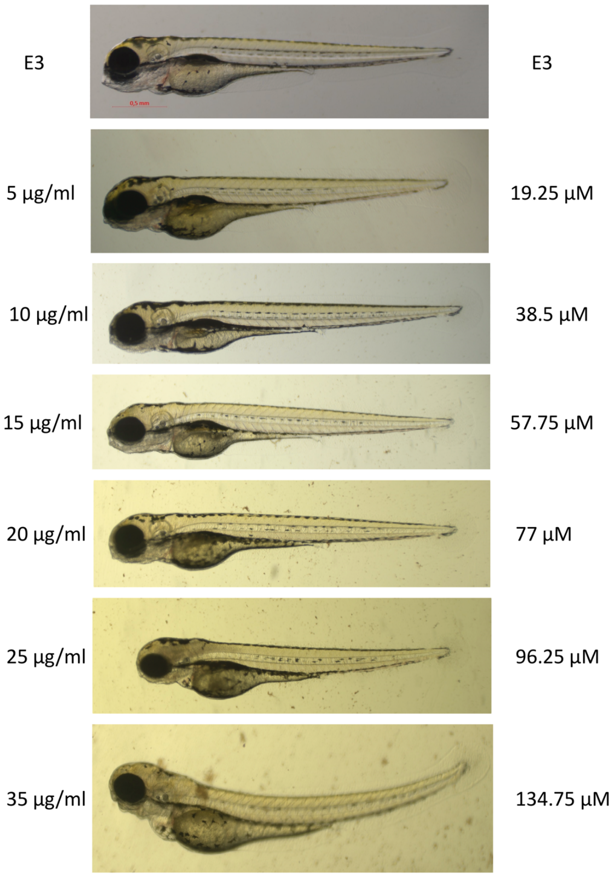

Figure S22. Images of *Danio rerio* exposed on ACF at the dose range: 5-35 µg/ml (19.25-96.2 µM) for 96 hours. Only slight developmental malformations (scoliosis, pericardial edema) were observed at the highest dose used, *i.e.*, 35 μg/ml (134.75 μM).

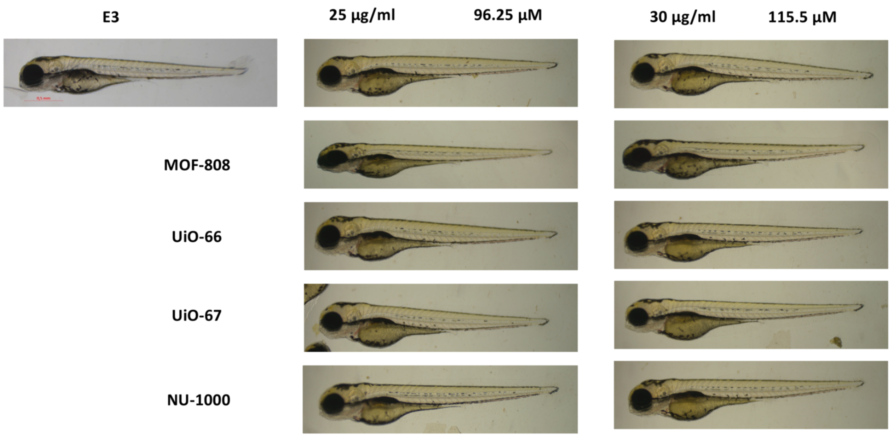

Figure S23. Comparison of 96h-long exposition of *Danio rerio* embryos on the solutions of ACF at concentrations of 25 and 30 μg/ml (96.25 and 115.5 μM) in form of free ACF or ACF released from the tested MOFs, *i.e.*, MOF-808, UiO-66, UiO-67, and NU-1000. No developmental malformations were observed.

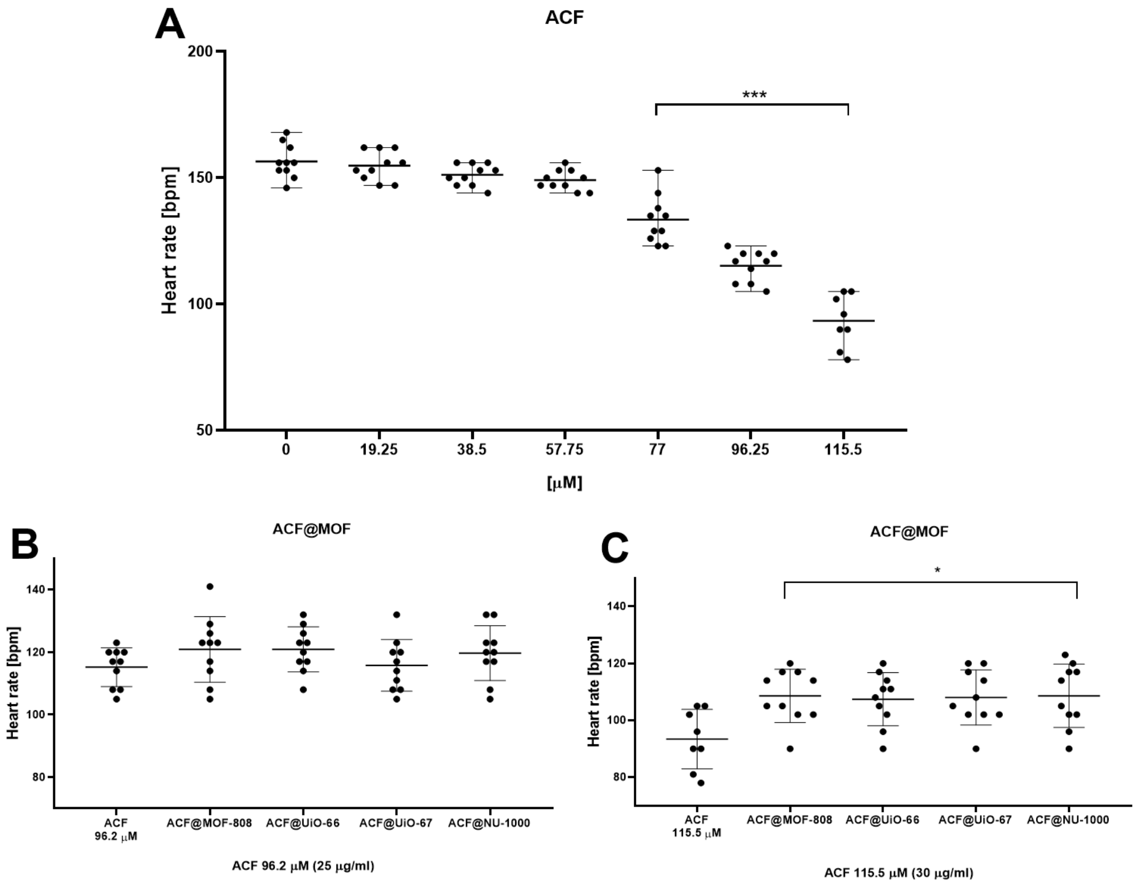

Figure S24. The cardiological effects of ACF and ACF@MOF in *Danio rerio* experimental model. (**A**) Dose dependent ACF effects on heart function of *Danio rerio* expressed as heart rate at 96 hpf. ***p<0.001 *vs* control 0 (E3) group, Tukey test. (**B**) Comparison of cardiological effect of free ACF at the dose of 25 μg/ml (96.2 μM) and the same dose of ACF released from MOF: 808, NU-1000, UiO-66, UiO-67 expressed as heart rate of *Danio rerio* at 96hpf. (**C**) Comparison of cardiological effect of free ACF at the dose of 30 μg/ml (115.5 μM) and the same dose of ACF released from MOF: 808, NU-1000, UiO-66, and UiO-67 expressed as heart rate of *Danio rerio* at 96 hpf. *p<0.05 *vs* free ACF group, Tukey test.

References

(1) Mondloch, J. E.; Bury, W.; Fairen-Jimenez, D.; Kwon, S.; Demarco, E. J.; Weston, M. H.; Sarjeant, A. A.; Nguyen, S. T.; Stair, P. C.; Snurr, R. Q.; Farha, O. K.; Hupp, J. T. Vapor-Phase Metalation by Atomic Layer Deposition in a Metal-Organic Framework. *J. Am. Chem. Soc.* **2013**, *135* (28), 10294–10297. https://doi.org/10.1021/ja4050828.

(2) Dai, S.; Simms, C.; Dovgaliuk, I.; Patriarche, G.; Tissot, A.; Parac-Vogt, T. N.; Serre, C. Monodispersed MOF-808 Nanocrystals Synthesized via a Scalable Room-Temperature Approach for Efficient Heterogeneous Peptide Bond Hydrolysis. *Chem. Mater.* **2021**, *33* (17), 7057–7066. https://doi.org/10.1021/acs.chemmater.1c02174.

(3) Jodłowski, P. J.; Kurowski, G.; Kuterasiński, Ł.; Sitarz, M.; Jeleń, P.; Jaśkowska, J.; Kołodziej, A.; Pajdak, A.; Majka, Z.; Boguszewska-Czubara, A. Cracking the Chloroquine Conundrum: The Application of Defective UiO-66 Metal–Organic Framework Materials to Prevent the Onset of Heart Defects—In Vivo and In Vitro. *ACS Appl. Mater. Interfaces* **2021**, *13*, 312–323. https://doi.org/10.1021/acsami.0c21508.

(4) Gutterød, E. S.; Lazzarini, A.; Fjermestad, T.; Kaur, G.; Manzoli, M.; Bordiga, S.; Svelle, S.; Lillerud, K. P.; Skúlason, E.; Øien-ØDegaard, S.; Nova, A.; Olsbye, U. Hydrogenation of CO2 to Methanol by Pt Nanoparticles Encapsulated in UiO-67: Deciphering the Role of the Metal-Organic Framework. *J. Am. Chem. Soc.* **2020**, *142* (2), 999–1009. https://doi.org/10.1021/jacs.9b10873.

(5) Sha, F.; Chen, Y.; Drout, R. J.; Idrees, K. B.; Zhang, X.; Farha, O. K. Stabilization of an Enzyme Cytochrome c in a Metal-Organic Framework against Denaturing Organic Solvents. *iScience* **2021**, *24* (6), 102641. https://doi.org/10.1016/j.isci.2021.102641.

(6) Kresse, G.; Hafner, J. Ab Initio Molecular Dynamics for Open-Shell Transition Metals. *Phys. Rev. B* **1993**, *48* (17), 13115–13118. https://doi.org/10.1103/PhysRevB.48.13115.

(7) Kresse, G.; Furthmüller, J. Efficiency of Ab-Initio Total Energy Calculations for Metals and Semiconductors Using a Plane-Wave Basis Set. *Comput. Mater. Sci.* **1996**, *6* (1), 15–50. https://doi.org/10.1016/0927-0256(96)00008-0.

(8) Blöchl, P. E. Projector Augmented-Wave Method. *Phys. Rev. B* **1994**, *50* (24), 17953–17979. https://doi.org/10.1103/PhysRevB.50.17953.

(9) Kresse, G.; Joubert, D. From Ultrasoft Pseudopotentials to the Projector Augmented-Wave Method. *Phys. Rev. B - Condens. Matter Mater. Phys.* **1999**, *59* (3), 1758–1775. https://doi.org/10.1103/PhysRevB.59.1758.

(10) Perdew, J. P.; Burke, K.; Ernzerhof, M. ERRATA: Generalized Gradient Approximation Made Simple. *Phys. Rev. Lett.* **1997**, *78* (7), 1396.

(11) Grimme, S. Accurate Description of van Der Waals Complexes by Density Functional Theory Including Empirical Corrections. *J. Comput. Chem.* **2004**, *25* (12), 1463–1473. https://doi.org/10.1002/jcc.20078.

(12) Grimme, S. Semiempirical GGA-Type Density Functional Constructed with a Long-Range Dispersion Correction. *J. Comput. Chem.* **2006**, *27* (15), 1787–1799. https://doi.org/https://doi.org/10.1002/jcc.20495.

(13) Yang, B.; Wheeler, J. I.; Sorensen, B.; Steagall, R.; Nielson, T.; Yao, J.; Mendez-Arroyo, J.; Ess, D. H. Computational Determination of Coordination Structure Impact on Adsorption and Acidity of Pristine and Sulfated MOF-808. *Mater. Adv.* **2021**, *2* (13), 4246–4254. https://doi.org/10.1039/d1ma00330e.

(14) Øien, S.; Wragg, D.; Reinsch, H.; Svelle, S.; Bordiga, S.; Lamberti, C.; Lillerud, K. P. Detailed Structure Analysis of Atomic Positions and Defects in Zirconium Metal-Organic Frameworks. *Cryst. Growth Des.* **2014**, *14* (11), 5370–5372. https://doi.org/10.1021/cg501386j.

(15) Liu, W.-G.; Truhlar, D. G. Computational Linker Design for Highly Crystalline Metal–Organic Framework NU-1000. *Chem. Mater.* **2017**, *29* (19), 8073–8081. https://doi.org/10.1021/acs.chemmater.7b01624.
